# Ferrocenoyl-adenines: substituent effects on regioselective acylation

**DOI:** 10.3762/bjoc.18.133

**Published:** 2022-09-19

**Authors:** Mateja Toma, Gabrijel Zubčić, Jasmina Lapić, Senka Djaković, Davor Šakić, Valerije Vrček

**Affiliations:** 1 Faculty of Pharmacy and Biochemistry, University of Zagreb, Ante Kovačića 1, 10000 Zagreb, Croatia; 2 Faculty of Food Technology and Biotechnology, University of Zagreb, Pierottijeva 6, 10000 Zagreb, Croatia

**Keywords:** DFT, ferrocene, nucleophilicity, purine, steric effect

## Abstract

A series of *N*^6^-substituted adenine–ferrocene conjugates was prepared and the reaction mechanism underlying the synthesis was explored. The S_N_2-like reaction between ferrocenoyl chloride and adenine anions is a regioselective process in which the product ratio (*N*7/*N*9-ferrocenoyl isomers) is governed by the steric property of the substituent at the *N*^6^-position. Steric effects were evaluated by using Charton (empirical) and Sterimol (computational) parameters. The bulky substituents may shield the proximal *N*7 region of space, which prevents the approach of an electrophile towards the *N*7 atom. As a consequence, the formation of *N*7-isomer is a kinetically less feasible process, i.e., the corresponding transition state structure increases in relative energy (compared to the formation of the *N*9-isomer). In cases where the steric hindrance is negligible, the electronic effect of the *N*^6^-substituent is prevailing. That was supported by calculations of Fukui functions and molecular orbital coefficients. Both descriptors indicated that the *N*7 atom was more nucleophilic than its *N*9-counterpart in all adenine anion derivatives. We demonstrated that selected substituents may shift the acylation of purines from a regioselective to a regiospecific mode.

## Introduction

Nucleosides in which the sugar part is replaced with an organometallic moiety have attracted remarkable interest [1–3]. One important class are ferrocene–nucleobase conjugates [4], which are known to exhibit anticancer [5–7], antibacterial [8–10], or antitrypanosomal activity [11], but also may serve as electrochemical biosensors [12–13], self-assembled molecular materials [14–15], decorations of carbon tubes and nanomaterials [16–17], or structural motifs in xeno nucleic acids [18].

In continuation of our work on ferrocenoyl-substituted pyrimidine nucleobases [19], we report herewith a combined theoretical and experimental work on purine series. The novelty of these compounds is the carbonyl linker which connects the organometallic (metallocene) and heterocyclic (purine) parts. Specifically, adenine and its *N*^6^-derivatives, most of which are pharmaceutically attractive and/or biologically relevant [20–22]*,* have been selected to study the mechanism underlying the synthesis of the ferrocene–nucleobase conjugates*.*

Several procedures for preparing *N*-ferocenoylated pyrimidines were tested earlier [19,23], and the reaction of nucleobase with (chlorocarbonyl)ferrocene (or ferrocenoyl chloride, FcCOCl) under basic conditions appeared as a simple and optimal method for the synthesis [24]. Herewith, we demonstrate that substituents at the exocyclic amino group of adenine affect the reactivity of the respective purine anion and govern the regioselectivity of the ferocenoylation reaction (*N*7- versus *N*9-product). By using an appropriate substituent at the *C*6 position in the purine ring, one can tune the isomeric product ratio, i.e. may influence the regioselectivity of the ferrocenoylation reaction. While the *N*9-position of the purine ring is a typical site of substitution, the *N*7-position may be preferred in some situations. In any case, the interplay between steric and electronic effects of selected substituents is crucial to kinetic and thermodynamic control of the acylation reaction.

## Results and Discussion

**Scheme 1:**
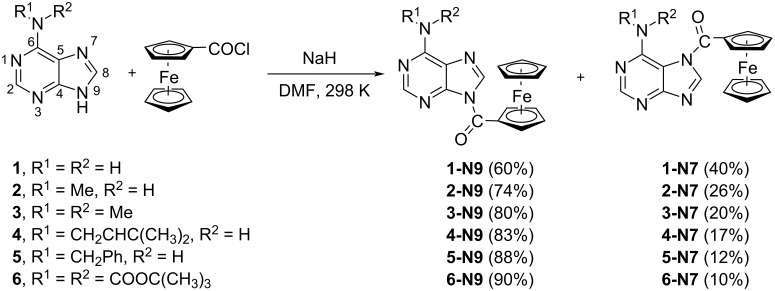
Regioselective ferrocenoylation of the adenine anion **1** and its derivatives **2**–**6** substituted at the *N*^6^-position. The respective NMR yields for regioisomers N9 and N7 are shown in parentheses.

It was shown that the reaction between the pyrimidine anion (uracil, thymine, or 5-fluorouracil) and FcCOCl in *N*,*N*-dimethylformamide (DMF) proceeded in a full regiospecific mode [19]. In the purine series, however, the analogous reaction is regioselective and resulted in the formation of two products, i.e., *N*7- and *N*9-regioisomers (Scheme 1). In no case, the *N*1-, *N*3-, or *N*^6^-products were formed, which is comparable to the results reported for the reaction between benzoyl chloride (BzCl) and purine anions [25].

**Figure 1:**
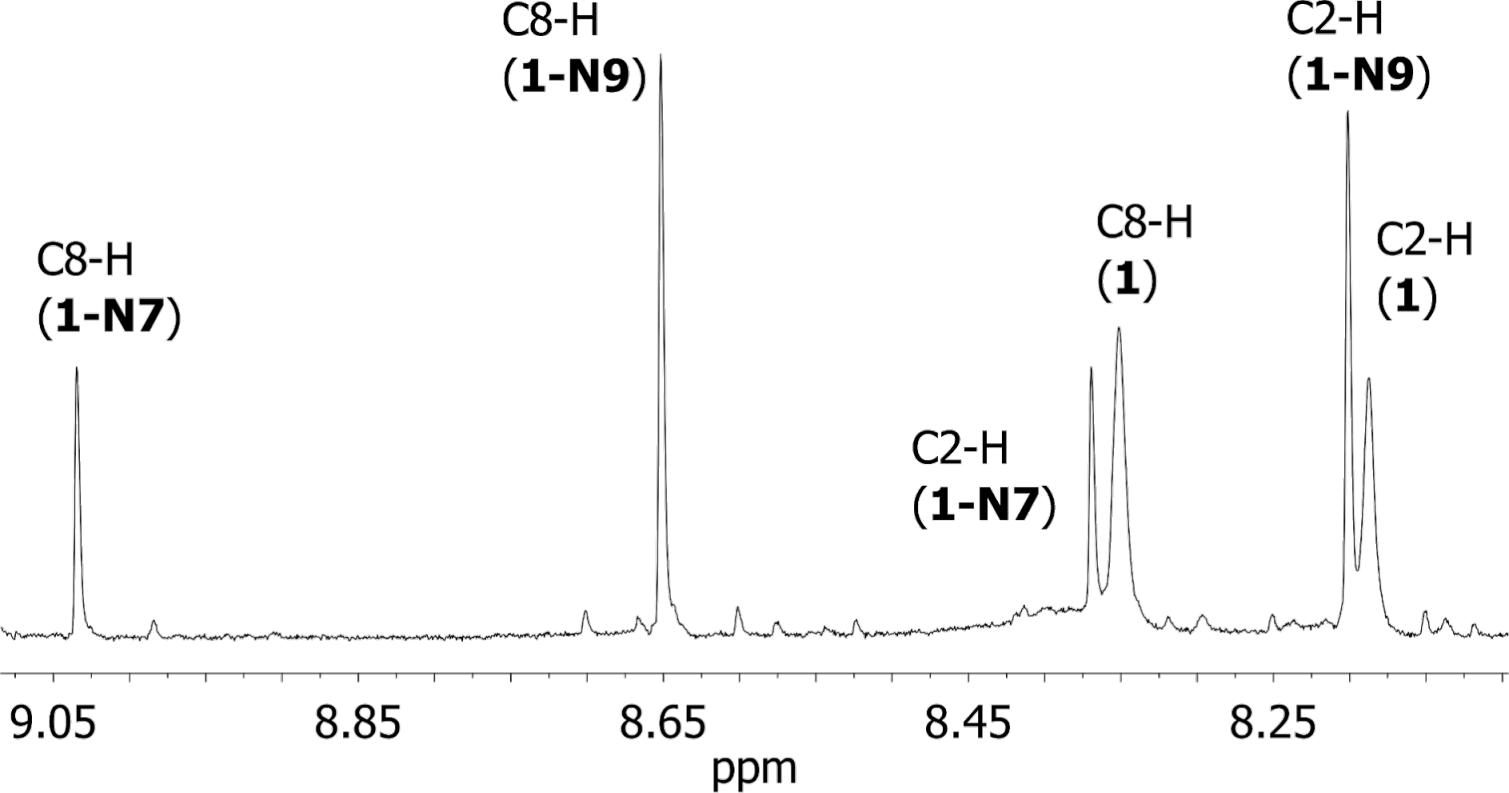
^1^H NMR spectrum (downfield region) of the reaction mixture (adenine anion **1** and FcCOCl) in DMF, taken after 10 minutes (see numbering in Scheme 1) at 25 °C. The DMF signal was suppressed by the presaturation option, and DMSO-*d*_6_ was used as a deuterated solvent in capillary.

The two different regioisomers, **1-N7** and **1-N9**, were formed simultaneously in the course of the reaction between adenine anion **1** and FcCOCl in DMF (Scheme 1). According to the ^1^H NMR spectrum (Figure 1) of a reaction aliquot, the ratio of *N*9/*N*7 isomers is 1.5:1, i.e. 60% regioselectivity is reached. The formation of both isomers is, therefore, a competitive process. This confirms that the adenine anion behaves as an ambident nucleophile with two competing reaction centers at the *N*7- and *N*9-position [26].

It is known that acylation [25] or alkylation [27–29] of adenine is rarely regiospecific, and mixtures of *N*7- and *N*9-isomers are usually obtained. In some cases, the acylation of adenine may also occur at the *exo*-amino group (*N*^6^) [30–31]. In general, the literature on the regioselectivity of alkylation of adenines/purines is more abundant, includes an array of reaction conditions (base, solvent, temperature) [32], and introduces various effects of microwaves [33], cyclodextrines [34], or tetrabutylammonium fluoride on the *N*7/*N*9-product ratio [35]. It is therefore of interest to collect complementary data on analogous acylation reactions, which are required for a future comparative study.

Intrinsic nucleophilicity [36] is an important factor in governing competition between the various nucleophilic centers in adenine. To estimate the relative inherent reactivity of different nucleophilic sites in the adenine anion, conceptual DFT tools were employed [37]. Specifically, frontier molecular orbital (FMO) properties and Fukui indices [38] were calculated to explain the observed regioselectivity in the ferrocenoylation of adenine.

**Figure 2:**
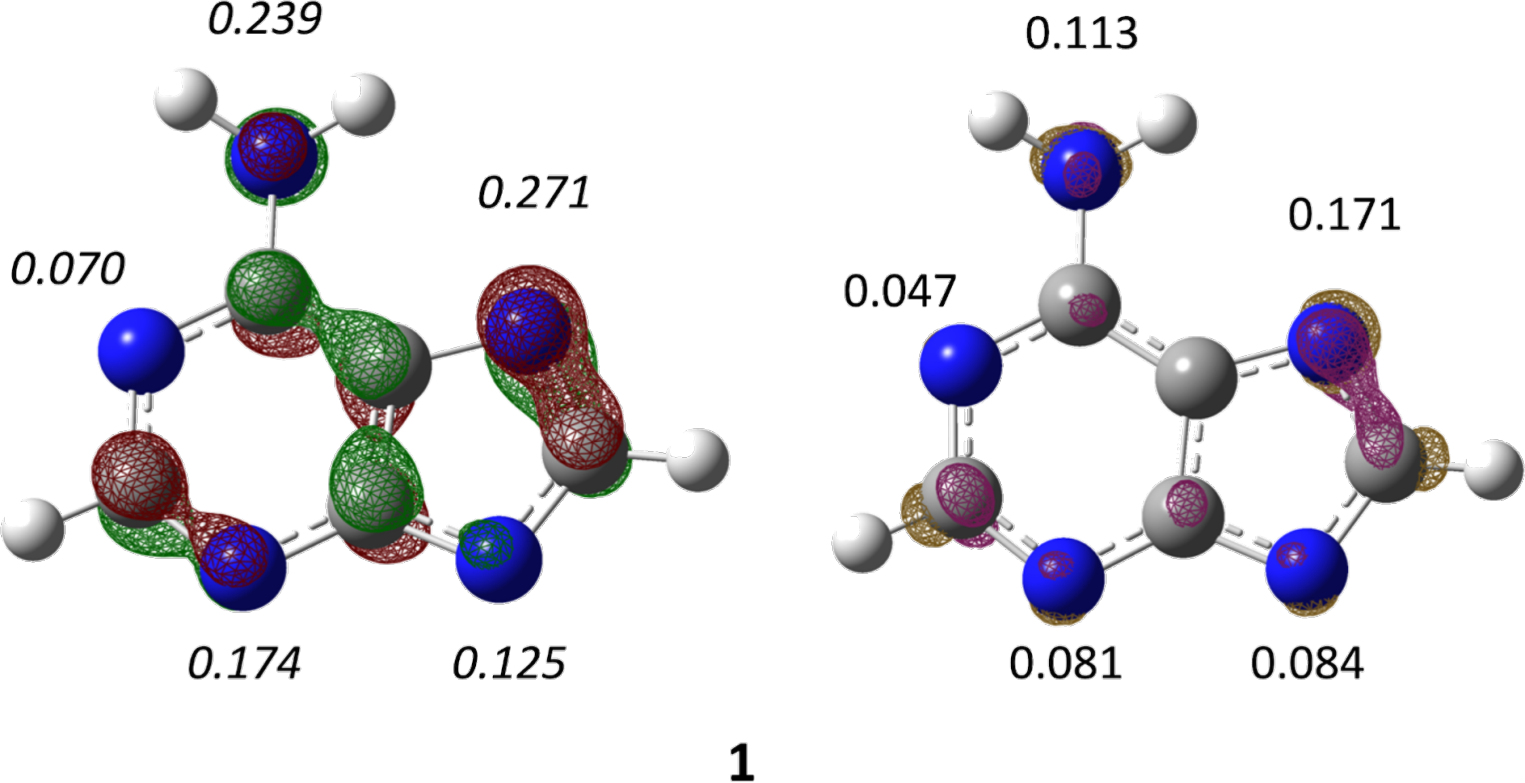
HOMO map of space distribution (left) of adenine anion (**1**) at the B3LYP/6-31+G(d) level of theory (MO = 35, isovalue = 0.08). Molecular orbital coefficients in italics. The isosurface plot of the condensed Fukui function *f*^-^ (right) for the adenine anion (isovalue = 0.008) calculated with NPA charges.

The visualization of the highest occupied molecular orbital (HOMO) of the adenine anion is useful in predicting the nucleophilic reactivity of different nitrogen atoms toward electrophilic substrates (Figure 2). The HOMO orbital is distributed over the purine ring with the largest amplitude on the *N*7 atom, which designates this position as the most nucleophilic in **1**. The second most populated site is the *N*^6^ atom, which appears as more nucleophilic than *N*9 atom.

To facilitate a quantitative comparison between different sites, the condensed Fukui function based on atomic charges was calculated. We calculated total populations for all nitrogen atoms in the adenine anion in its N and N−1 electron states to obtain the condensed *f*^−^ descriptor according to the equation for the nucleophilicity (for details, see Computational part in Supporting Information File 1).

In purines **2**–**5**, the method for charge fitting suggests that the most positive part of the *f*^–^ function is localized at the *N*^6^ atom, which means that this nitrogen is the most nucleophilic site in adenines (except **1** and **6**). In no case, however, the ferrocenoylation reaction at the *N*^6^ position was observed in ^1^H NMR spectra. This is expected, as the nucleophilic addition pathway involving the quaternary ammonium intermediate is not viable.

In all purine anions, according to the calculated Fukui functions *f*^–^, the *N*7 atom is more nucleophilic than the *N*9 atom. It comes out that the *N*7-nitrogen in the adenine anion reacts more readily with electrophiles, i.e., nucleophilic reactions occur preferably at the *N*7-position. The same conclusion was made by Stachowicz–Kuśnierz and Korchowiec who have shown that the inherent nucleophilicity of the *N*7 atom is higher compared to that of the *N*9 atom [39].

This is, however, in discrepancy with the regioselectivity observed in the ^1^H NMR spectra (Figure 1) of the reaction mixture, where the formation of the *N*9-isomer (**1-N9**) is favored. This suggests that intrinsic nucleophilicity or Fukui functions are not sufficient to explain the regioselective reaction, but other factors are to be considered.

The disagreement between the experimentally observed *N*9/*N*7 regioselectivity and calculated *N*9/*N*7 nucleophilicity was found (Table 1) for all adenine derivatives in which the *N*^6^ atom was substituted with different groups (H, Me, Bz, isopentenyl, or Boc). In **1** and **6** the *N*7 nitrogen atom was calculated the most nucleophilic (Table 1, values in bold), suggesting this site should be acylated predominantly. On the contrary, according to ^1^H NMR analysis and isolated yields, the *N*9-isomer was the major product in each case. It comes out that electronic properties of the respective purine are not decisive in terms of regioselectivity. Instead, steric effects may govern the *N*9/*N*7 ratio in the reaction mixture.

**Table 1:** The condensed Fukui functions *f*^−^ (based on NBO atomic charges) for nitrogen atoms in the purine anions calculated at the (U)B3LYP/6-31+G(d) method.^a^

Purine anion	Nitrogen atom					Exp. ratio^b ^*N*9:*N*7

	*N*1	*N*3	*N* ^6^	*N*7	*N*9	

**purine** ^c^	0.0460	0.0691	–	**0.2730**	**0.1611**	1:2.4
**1**	0.0468	0.0805	0.1131	**0.1705**	0.0836	1.5:1
**2**	0.0411	0.0726	**0.1351**	0.1339	0.0727	2.8:1
**3**	0.0389	0.0771	**0.1590**	0.1223	0.0704	4.0:1
**4**	0.0418	0.0706	**0.1403**	0.1303	0.0696	4.9:1
**5**	0.0449	0.0721	**0.1424**	0.1260	0.0682	7.3:1
**6**	0.0364	0.0561	0.0120	**0.2483**	0.1338	9.0:1

^a^The largest value of *f*^−^ in the respective purine anion is in bold; ^b^isomer ratio determined from the ^1^H NMR spectrum of the corresponding reaction mixture in DMF; ^c^the anion derived from the parent 9*H*-purine structure.

Attempts to find a correlation between the *N*9/*N*7 ratio and the steric bulk of the *C*6-substituent in the alkylation of purines were reported earlier [40]. Now, we demonstrate for the first time that similar effect is operative in the acylation of purines. It is evident from the results in Table 1 that the *N*9/*N*7 ratio increases with the increasing size of the substituent at the exocyclic amino group.

In case when the steric effect is negligible (e.g., H atom at the *C*6-position), the *N*9-isomer is a minor product (less than 30%), and the acylation of the *N*7 position is strongly favored. In the parent purine, therefore, the nucleophilic attack is mostly controlled by electronic effects, i.e., the regioselectivity is governed by the intrinsic nucleophilicity of the *N*7 position (Table 1).

**Figure 3:**
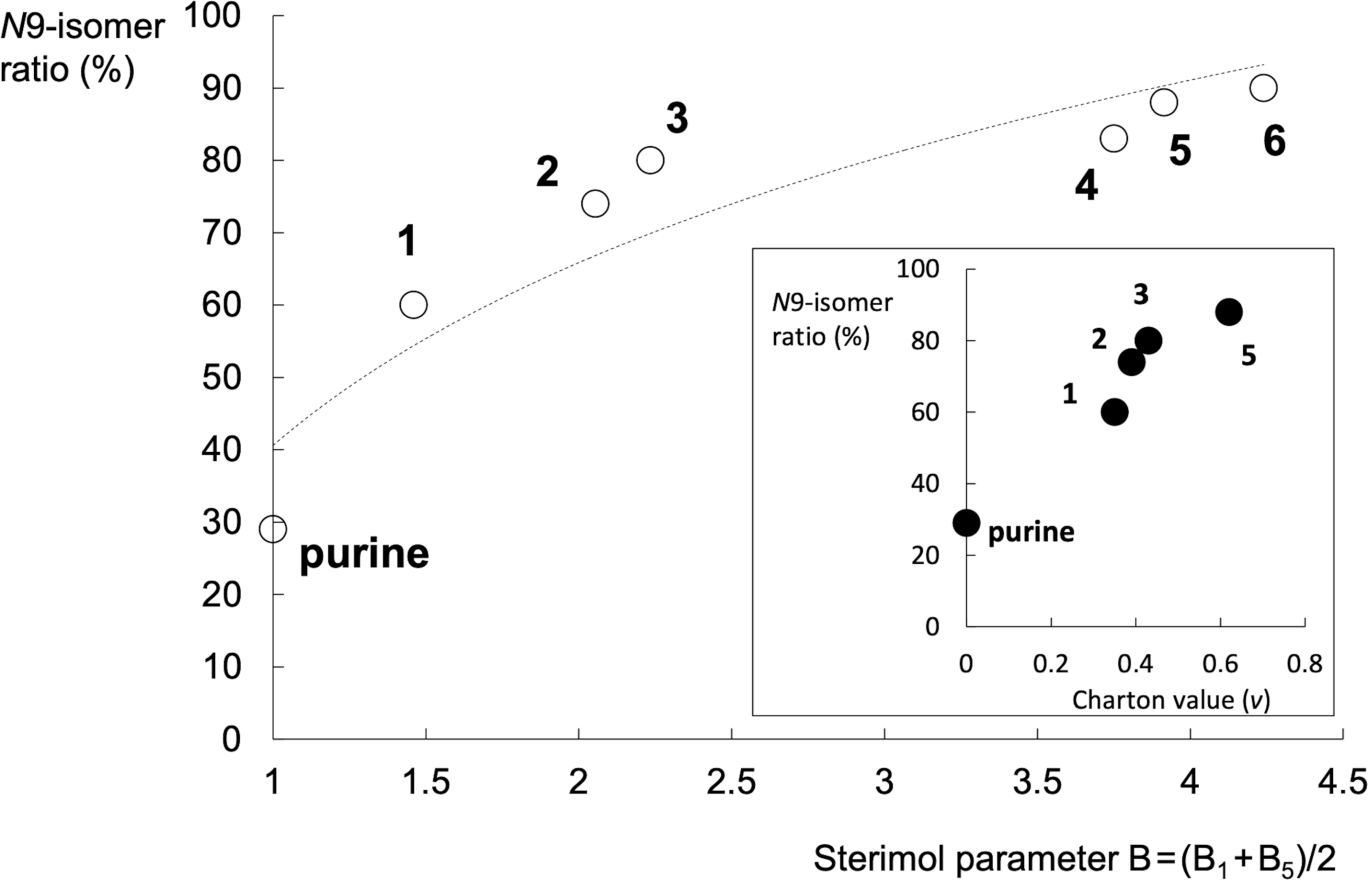
Dependence of *N*9-isomer product ratio (%), in the reaction between FcCOCl and adenine anions **1**–**6**, on the averaged perpendicular Sterimol parameter B = (B_1_ + B_5_)/2 of the *C*6-substituent (open circles; in the parent purine the *C*6-substituent is a H atom). The logarithmic trendline is for illustration purposes only. The inset shows the dependence of *N*9-isomer ratio (%) on the Charton value (closed circles; ν is not available for all *C*6-substituents). Purine (B = 1; ν = 0) is the anion derived from the parent 9*H*-purine structure.

To correlate steric effects in ferrocenoylation reactions of purines to the measured *N*9/*N*7 ratio, the Charton (ν) [41–42] and Sterimol steric parameters [43–45] for selected substituents were introduced (Figure 3). The former parameter is empirical, and is not available for all functional groups, while the latter is a computational parameter, which constitutes a significant improvement in terms of overall utility and accuracy [43].

An increasing trend of the *N*9/*N*7 ratio with steric bulk was observed in both cases (Figure 3). However, Charton parameters for isopentenyl (as in **4**) and Boc group (as in **6**) substituents (to name but a few) do not exist in the literature, thus limiting the number of points and the range of values in our diagram. Sterimol parameters are broadly applicable Boltzmann-weighted parameters, which are conformationally dependent, and thus may define the steric effect for any conceivable substituent. They are multidimensional parameters, that is, they describe steric bulk along different principal axes, hence, the effects of unsymmetrical substituents are better described with Sterimol parameters. B_1_, B_5_, and L_0_ subparameters comprise Sterimol parameters and are defined using Corey–Pauling–Koltun (CPK) molecular models, with B_1_ being the shortest perpendicular distance from the primary axis of the attachment, B_5_ being the maximum width form the same axis and L_0_ representing the total distance along the primary axis of the attachment. All Boltzmann-weighted subparameters for all purines (**1**–**6**) are deposited in Table S2 (Supporting Information File 1).

In addition, we calculated the percentage of buried volume (%V_Bur_), another popular steric descriptor which may be applied to quantify the fraction of the defined sphere around a reaction center [46]. It was introduced for ligands on metals [47], but may be adapted to estimate the steric hindrance of substituents in different chemical environments (see Supporting Information File 1 for more details). As expected, within the group of *N*7-regioisomers, the calculated %V_Bur_ increases with more bulkier groups at the C6-position (as going from **1** to **6**), whereas no significant effects are observed in the series of *N*9-isomers (Table S3 in Supporting Information File 1). These results nicely complement the trend measured with Sterimol parameters.

We assume that the steric effect of the *C*6-substituent is the most evident in the transition state structure leading to the formation of the *N*7-ferrocenoylated product. The bulky substituents at the *C*6 atom may shield the proximal *N*7 region of space, which prevents the approach of an electrophile (e.g., FcCOCl) towards the *N*7 atom. In the course of *N*9-isomer formation no similar steric hindrance is encountered.

**Figure 4:**
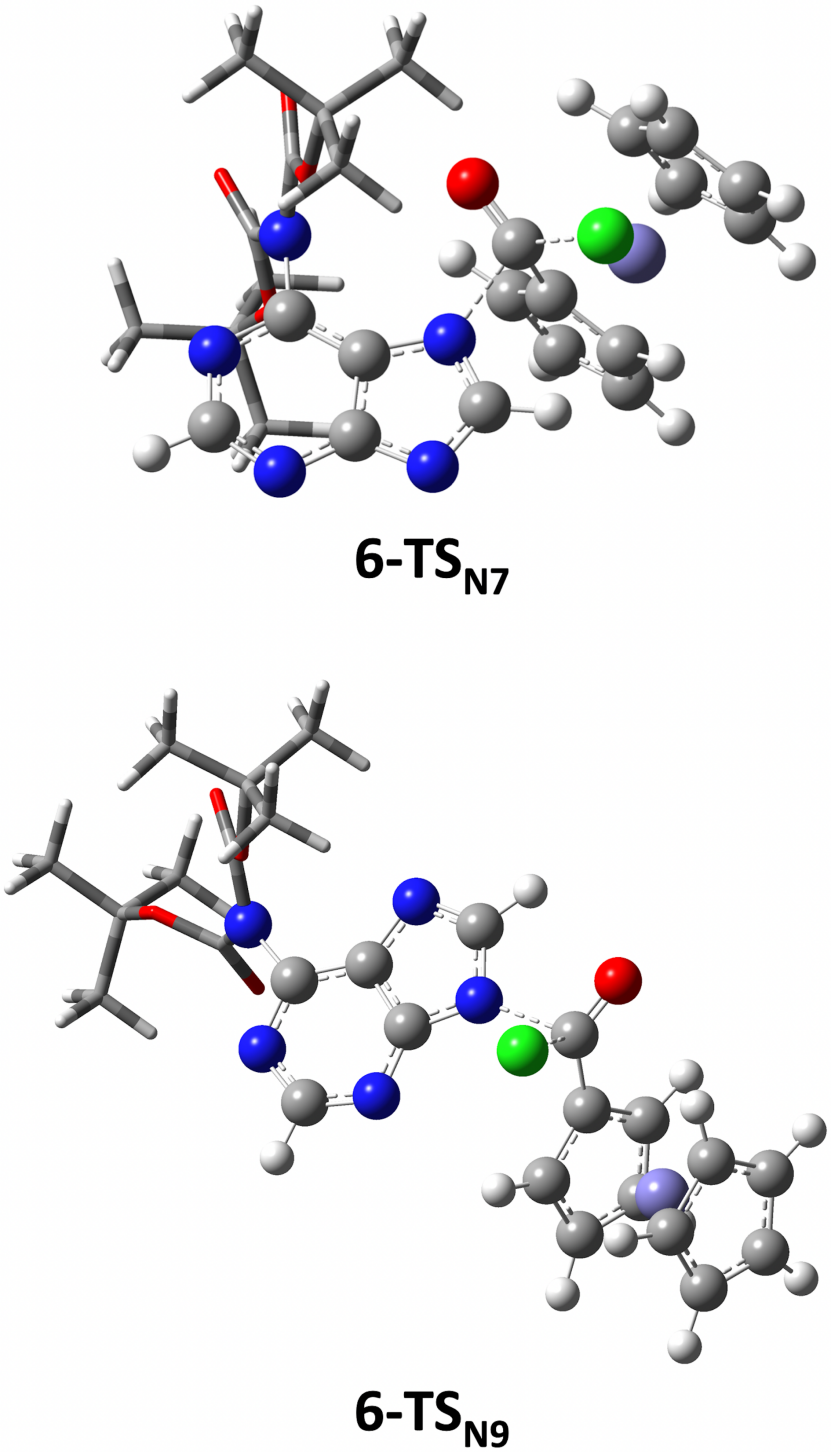
B3LYP/6-31+G(d)/SDD optimized transition state structures for *N*7- (**6-TS_N7_**) and *N*9-ferrocenoylation (**6-TS_N9_**) of the adenine anion **6**. Two bulky *tert*-butyloxycarbonyl groups are displayed in the tube mode for clarity.

This is supported by our quantum-chemical calculations which compared the two transition state structures for the ferrocenoylation of the *N*^6^,*N*^6^-di-*tert*-butyloxycarbonyladenine, i.e., the derivative with the bulkiest substituents at the C6-position. The calculated energy barrier for the formation of the *N*7-isomer (**6-N7**) is higher than the corresponding barrier for the formation of the *N*9-isomer (**6-N9**) (ΔΔ*G*^‡^ = 11.3 kJ/mol). The respective transition state structures **6-TS_N7_** and **6-TS_N9_** (Figure 4) are characterized by one imaginary frequency (134*i* and 163*i* cm^−1^, respectively), which corresponds to the N–C bond formation concomitant with C–Cl bond breaking. Both structures support a concerted S_N_2-type mechanism in which a tetrahedral intermediate does not exist. Therefore, the one-step mechanism is operative in the reaction between adenine anion **6** and FcCOCl.

The structure **6-TS_N7_** is characterized with an unfavorable steric repulsion between the *tert*-butyloxycarbonyl group at the C6-position and the acyl group approaching the *N*7 atom. This steric constraint is not present in the transition structure **6-TS_N9_**, which is, as reported above, more stable (11.3 kJ/mol) than the structure **6-TS_N7_**. It comes out that the regioselectivity of the ferrocenoylation of adenine anion **6** is kinetically controlled, mostly due to steric effects.

The same S_N_2-type mechanism is operative for the reaction between *N*^6^-substituted adenine anions **1**–**5** and FcCOCl. In no case the tetrahedral intermediate, typical of a nucleophilic addition–elimination pathway, was located as a genuine minimum on the potential energy landscape. Instead, the structure with tetrahedral geometry corresponds to the transition state, which directly (in a single step manner) connects respective reactants and acylated product. All optimized geometries are deposited in Supporting Information File 1.

**Figure 5:**
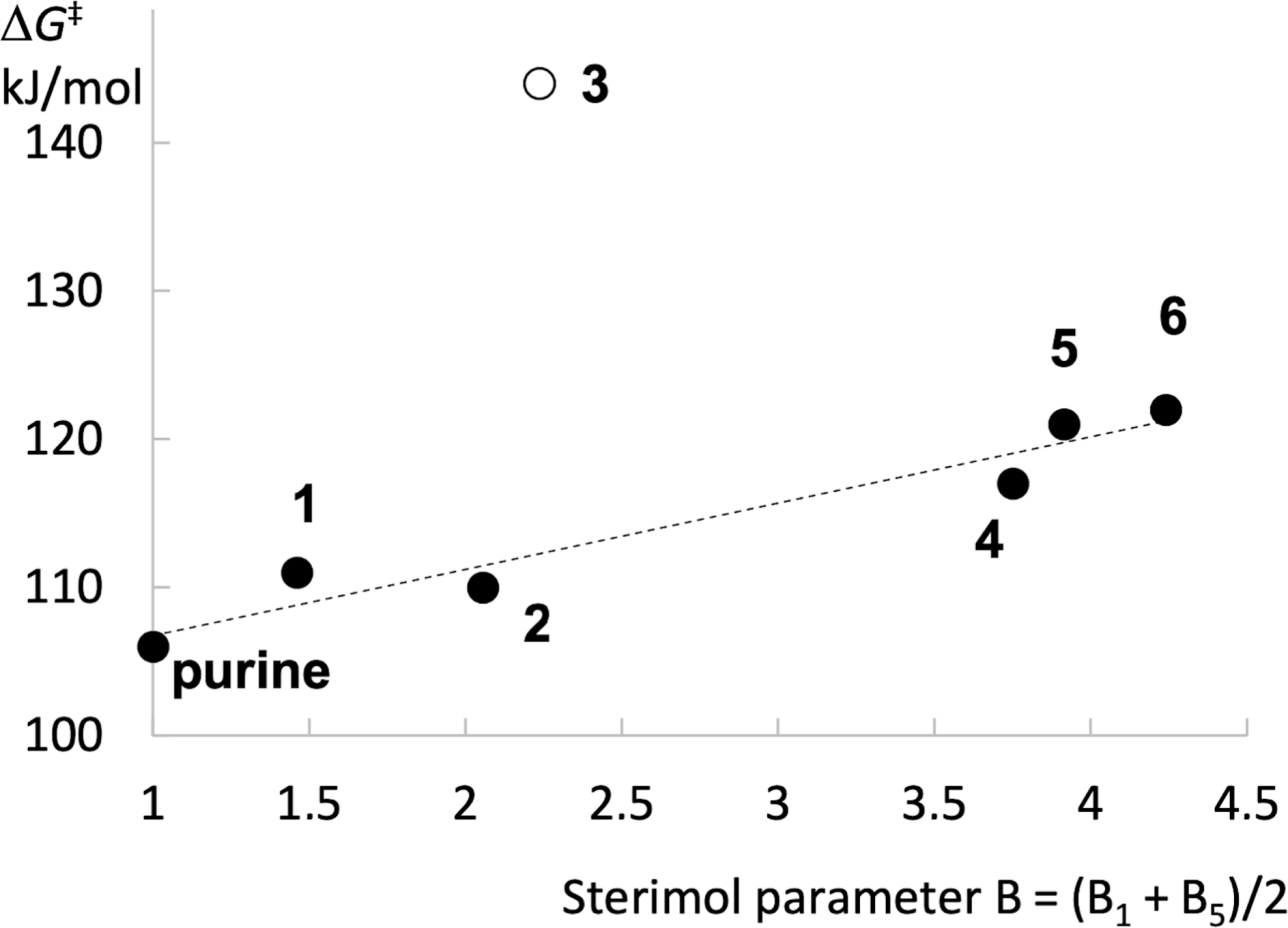
Relation between the Gibbs free energy barrier (Δ*G*^‡^) for the *N*7-ferrocenoylation of C6-substituted adenines and the averaged perpendicular Sterimol parameter B = (B_1_ + B_5_)/2. The linear trendline is for illustration purposes only. Data point for **3** (*N*^6^,*N*^6^-dimethyladenine; open circle) appears as an outlier. Purine (B = 1) is the anion derived from the parent 9*H*-purine structure.

According to the calculated results, the energy barrier for the *N*7-ferrocenoylation reaction increases with the size of the group attached at the C6 position (Figure 5). A nearly linear relationship (*r*^2^ = 0.93) between the calculated barrier (Δ*G*^‡^) for the *N*7-ferrocenoylation and the steric parameter (B) is obtained. The only exception is the energy barrier for the *N*7-ferrocenoylation of the *N*^6^,*N*^6^-dimethyladenine (**3**). For some reason, the calculated barrier is prohibitively high (Δ*G*^‡^ = 144 kJ/mol), which suggests an inappropriate quality of the selected theoretical level, or indicates that an alternative ferrocenoylation mechanism in case of **3** is operative (e.g., the reaction which includes the quaternary nitrogen intermediate, see Scheme S1 in Supporting Information File 1). In any case, the *N*7-ferrocenoylation of adenine anion **3** is a viable process, as demonstrated by the ^1^H NMR spectroscopy evidence (see above), and by the isolation of the *N*7-ferrocenoylated product.

We have continued our study with an extended set of *C*6- and *C*2-substituents in the purine ring, but preliminary results suggested that a simple correlation between the *N*9/*N*7 product ratio and steric parameters was lost. In order to relate the regioselectivity observed in the acylation of purines, additional descriptors, such as the Swain and Lupton resonance parameter, may be included in multiple regression analysis [40]. In some cases, e.g., *C*6-chloropurine and *C*6-bromopurine, only the *N*9-ferrocenoylated product was detected (see Figures S3 and S4 in Supporting Information File 1), which confirms that the regioselective reaction may be switched to the regiospecific mode by selecting suitable substituents on the purine ring.

## Conclusion

In the reaction between *N*^6^-substituted adenine anions and ferrocenoyl chloride two regioisomeric products were formed: *N*7- and *N*9-ferrocenoylated adenines. The product ratio is strongly dependent on the steric parameter of the *N*^6^-substituent. The *N*9/*N*7 ratio is increasing with the larger substituent size. The bulkiness of substituents (H, Me, Bz, isopentenyl, and *tert*-butyloxycarbonyl) was defined by Charton and/or Sterimol parameters. The latter descriptor is a computational descriptor and may be assigned to any type of substituents. Specifically, the averaged perpendicular Sterimol parameter B linearly correlate with the calculated energy barrier (Δ*G*^‡^) for the *N*7-ferrocenoylation of adenine derivatives, which supports our claim that the observed *N*9/*N*7 regioselectivity is kinetically controlled.

When steric hindrance is negligible (e.g., the parent purine), the regioselectivity for a respective reaction is governed by electronic properties, such as nucleophilicity and/or electrophilicity. This may be predicted computationally using the conceptual DFT approach. We applied Fukui indices as descriptors for chemical reactivity, which revealed that the *N*7 atom is more nucleophilic than the *N*9 atom in all adenine derivatives. Both, steric and electronic properties are to be included when considering the regioselectivity of the acylation reaction of purines. In some cases, the regioselectivity was translated into a regiospecific process, i.e., only the *N*9-regioisomer appeared as a product in the ferocenoylation reaction.

## Supporting Information

File 1 Details on experimental procedures, DFT calculated energies and optimized coordinates for transition state structures and reactants, and the results of in situ ^1^H NMR monitoring.
