# Supplementary material for: Ferrocenoyl-adenines: substituent effects on regioselective acylation
Source: Beilstein J Org Chem. 2022 Sep 19;18:1270–7. doi: 10.3762/bjoc.18.133 (PMC9520867; doi:10.3762/bjoc.18.133)
Supplement: File 1 — Details on experimental procedures, DFT calculated energies and optimized coordinates for transition state structures and reactants, and the results of in situ 1H NMR monitoring. [file Beilstein_J_Org_Chem-18-1270-s001.pdf]

BEILSTEIN JOURNAL OF ORGANIC CHEMISTRY

Supporting Information

for

Ferrocenoyl-adenines: substituent effects on regioselective acylation

Mateja Toma, Gabrijel Zubčić, Jasmina Lapić, Senka Djaković, Davor Šakić and Valerije Vrček

Beilstein J. Org. Chem. **2022,** *18,* 1270–1277. doi:10.3762/bjoc.18.133

Details on experimental procedures, DFT calculated energies and optimized coordinates for transition state structures and reactants, and the results of in situ ^{1}H NMR monitoring

License and Terms: This is a supporting information file under the terms of the Creative Commons Attribution License (https://creativecommons.org/licenses/by/4.0). Please note that the reuse, redistribution and reproduction in particular requires that the author(s) and source are credited and that individual graphics may be subject to special legal provisions.
The license is subject to the *Beilstein Journal of Organic Chemistry* terms and conditions: (https://www.beilstein-journals.org/bjoc/terms)

Table of contents

1. Experimental part S2

1.1. Methods and materials S2

1.2. Preparation of ferrocenoylated adenines S2

1.3. In situ ^{1}H NMR monitoring of the reaction between adenine anions and FcCOCl S8

1.4. Selected ^{1}H NMR spectra of the reaction mixture (regiospecific mode) S10

2. Computational part S11

2.1. Computational techniques and methods S11

2.2. Calculated energies and barriers S12

2.3. S_N2-type and alternative reaction mechanisms for *N*7-ferocenoylation of adenines S13

2.4. Calculation of Sterimol and % V_{Bur} parameters S14

2.5. Optimized coordinates of reactants and transition states S17

3. References S31

1. Experimental part

1.1. Methods and materials

The syntheses were carried out under argon atmosphere in dry solvents. The IR spectra were recorded in chloroform with a Cary 630 FTIR Spectrometer (Agilent). The mass spectra were acquired using 4800 MALDI TOF/TOF-MS Analyzer. The ^{1}H and ^{13}C NMR spectra were recorded at 25 °C on a Varian INOVA 400 spectrometer. The spectrometer operated at 399.6 MHz (^{1}H) and 100.5 MHz (^{13}C). Chemical shifts in the ^{1}H NMR and ^{13}C NMR spectra were expressed in parts per million (ppm) and referenced to the residual solvent resonances. Products were purified by column chromatography (Fluka, Silica gel, 90 Å, 70–230 mesh) using the mixtures $CH_2Cl_2/(CH_3)_2CO$ 10:1.

1.2. Preparation of ferrocenoylated adenines

A detailed protocol for the preparation and characterization of ferrocenoylated purines was described earlier.[1] Some of ferrocenoyl-adenines (**1-N7**, **1-N9**, **5-N7**, and **5-N9**) have been reported earlier,[1] and therefore only ^{1}H and ^{13}C NMR spectra are included herewith. In short, sodium hydride, NaH (1.5 mmol), was added portionwise to purine bases (1 mmol) suspended in 3 mL DMF. After stirring at room temperature for 30 min, FcCOCl (1 mmol) was added dropwise to the clear solution. The mixture was stirred for 15–30 minutes and then was neutralized with 10% aqueous solution of citric acid and extracted with CH_2Cl_2. The organic layer was washed with water and evaporated under vacuum. A purification by column chromatography (silica gel, dichloromethane/acetone 10:1) afforded *N7*- or *N*9-ferrocenoylated adenines.

*N*7-Ferrocenoyl-adenine (**1-N7**); NMR spectra in agreement with those reported earlier[1]: ^{1}H NMR (400 MHz, DMSO-d$_6$) δ/ppm: 9.00 (s, 1H), 8.37 (s, 1H), 7.07 (s, 2H), 5.11 (pt, 2H), 4.86 (pt, 2H), 4.34 (s, 5H). ^{13}C{1H} NMR (100 MHz, DMSO-*d*$_6$) δ/ppm: 171.09, 161.07, 155.04, 152.63, 146.65, 109.55 74.37, 72.65, 71.35, 69.92.

*N*9-Ferrocenoyl-adenine (**1-N9**); NMR spectra in agreement with those reported earlier[1]: ^{1}H NMR (400 MHz, DMSO-d$_6$) δ/ppm: 8.65 (s, 1H), 8.19 (s, 1H), 7.48 (s, 2H), 5.02 (pt, 2H), 4.78 (pt, 2H), 4.31 (s, 5H). ^{13}C{1H} NMR (100 MHz, DMSO-*d*$_6$) δ/ppm: 169.73, 156.82, 154.07, 149.61, 139.88, 119.25, 74.10, 72.63, 71.04, 69.92.

*N*7-Ferrocenoyl-*N*^{6}-methyladenine (**2-N7**): ^{1}H NMR (400 MHz, $CDCl_3$, 25 °C) δ/ppm: 8.85 (s, 1H, C8−H), 8.64 (s, 1H, C2−H), 7.37 (s, 1H, NH), 4.97 (pt, 2H, Cα-H), 4.77 (pt, 2H, Cβ-H), 4.33 (s, 5H, Cp-H), 3.23 (s, 3H, CH_3). ^{13}C{^{1}H} NMR (100 MHz, $CDCl_3$, 25 °C) δ/ppm: 171.38 (FcCO), 160.03 (C2), 155.49 (C6), 152.17 (C4), 144.23 (C8), 110.38 (C5), 73.83 (Cβ), 72.24 (Cα), 71.12 (Cp), 69.96 (Ci), 28.07 (CH_3).

*N*9-Ferrocenoyl-*N*^{6}-methyladenine (**2-N9**): ^{1}H NMR (400 MHz, $CDCl_3$, 25 °C) δ/ppm: 8.49 (s, 1H, C8−H), 8.37 (s, 1H, C2−H), 6.31 (s, 2H, NH_2), 5.07 (pt, 2H, Cα-H), 4.68 (pt, 2H, Cβ-H), 4.26 (s, 5H, Cp-H), 3.32 (s, 3H, CH_3). ^{13}C{^{1}H} NMR (100 MHz, $CDCl_3$, 25 °C) δ/ppm: 171.16 (FcCO), 169.26 (C2), 155.68 (C6), 154.46 (C4), 138.75 (C8), 120.10 (C5), 73.59 (Cβ), 72.50 (Cα), 70.85 (Cp), 69.92 (Ci), 27.52 (CH_3).

*N*7-Ferrocenoyl-*N*^{6}, *N*^{6}-dimethyladenine (**3-N7**): red crystals (44.7 mg, 11% isolated yield (1.0 mmol, 163.2 mg of dimethyladenine), mp > 200 °C. ^{1}H NMR (400 MHz, DMSO-*d*$_6$, 25 °C)

δ/ppm: 8.90 (s, 1H, C8-H), 8.85 (s, 1H, C2-H), 5.26 (pt, 2H, Cα-H), 5.14 (pt, 2H, Cβ-H), 4.69 (s, 5H, Cp-H), 3.30 (s, 6H, 2xCH_3). $^{13}C\{^1H\}$ NMR (100 MHz, DMSO-d_6, 25 °C) δ/ppm: 171.77 (FcCO), 165.97 (C6), 161.22 (C8), 152.24 (C4), 141.51 (C2), 110.71 (C5), 73.21 (Cβ), 72.55 (Cα), 71.63 (Ci), 71.01 (Cp), 37.45 (CH_3). IR (CH_2Cl_2) ν_{max}/cm^{-1}: 3100 (w C-H), 3068 and 2943 (w C−H and =C−H, Fc), 1677 (s and w C=O), 1599 (s, stretching of purine ring), 1500-1200 (C=C and C=N stretching). UV–vis (CH_2Cl_2, 25 °C) λ/nm: 465. ε/dm^3 mol^{-1} cm^{-1}: 6.1 x 10^2. HRMS (MALDI-TOF/TOF) *m*/z: $[M + H]^+$ calculated for $C_{18}H_{17}FeN_5O$, 376.0816; found 376.0873.

*N*9-Ferrocenoyl-N^6,N^6-dimethyladenine (**3-N9**): red crystals (223.4 mg, 55% isolated yield (1.0 mmol, 163.2 mg of dimethyladenine), mp > 200 °C. *N*9 isomer was isolated from the reaction mixture and purified twice by column chromatography with the eluent dichloromethane/acetone 7:1.

^1H NMR (400 MHz, DMSO-d_6, 25 °C) δ/ppm: 8.46 (s, 1H, C8-H), 8.25 (s, 1H, C2-H), 4.98 (pt, 2H, Cα-H), 4.78 (pt, 2H, Cβ-H), 4.32 (s, 5H, Cp-H), 3.38 (s, 6H, 2xCH_3). $^{13}C\{^1H\}$ NMR (100 MHz, DMSO-d_6, 25 °C) δ/ppm: 169.78 (FcCO), 154.87 (C6), 153.25 (C8), 150.54 (C4), 138.50 (C2), 119.70 (C5), 74.22 (Cβ), 72.56 (Cα), 71.83 (Ci), 71.04 (Cp), 38.55 (CH_3). IR (CH_2Cl_2) ν_{max}/cm^{-1}: 3103 (w C-H), 3047 and 2931 (w C−H and =C−H, Fc), 1702 (s and w C=O), 1599 (s, stretching of purine ring), 1500-1200 (C=C and C=N stretching). UV–vis (CH_2Cl_2, 25 °C) λ/nm: 464. ε/dm^3 mol^{-1} cm^{-1}: 5.9 × 10^2. HRMS (MALDI-TOF/TOF) *m*/z: $[M + H]^+$ calculated for $C_{18}H_{17}FeN_5O$, 376.0816; found 376.0874.

*N*7-Ferrocenoyl-N^6-(2-*iso*pentenyl)adenine (**4-N7**): red crystals (39.3 mg, 9% isolated yield (1.0 mmol, 203.2 mg of 2-*iso*pentenyladenine), mp > 200 °C. *N*7 and *N*9 isomers **4** were

separated by column chromatography with eluent dichloromethane/acetone 10:0.9 whereby *N*7 isomer was additionally purified on a column with the eluent dichloromethane/acetone 7:1.

^{1}H NMR (400 MHz, $CDCl_3$, 25 °C) δ/ppm: 8.80 (s, 1H, C8-H), 8.62 (s, 1H, C2-H), 7.18 (t, broad, 1H, NH), 5.40 (t, J = 7,0 Hz, 1H, CH=), 4.98 (pt, 2H, Cα-H), 4.77 (pt, 2H, Cβ-H), 4.32 (s, 5H, Cp-H), 4.19 (dd, J = 6.5 and 5.6 Hz, 2H, CH_2), 1.76 (dd, J = 1.4 and 0.9 Hz, 6H, 2xCH_3). $^{13}C\{^1H\}$ NMR (100 MHz, $CDCl_3$, 25 °C) δ/ppm: 171.23 (FcCO), 169.51, 160.22 (C4), 155.49 (C2). 151.29 (C6), 144.10 (C8), 136.81 (C=), 120.07 (CH=), 110.30 (C5), 73.85 (Cβ), 72.25 (Cα), 71.52 (Ci), 71.12 (Cp), 39.09 (CH_2), 25.69 (CH_3), 18.05 (CH_3). IR (CH_2Cl_2) ν_{max}/cm^{-1}: 3380-3204 (w NH), 2976 (w C−H and =C−H, Fc), 1681 (s, C=O), 1614 (s, stretching of purine ring), 1500-1200 (C=C and C=N stretching). UV–vis (CH_2Cl_2, 25 °C) λ/nm: 471.9; 349.0 ($c = 1 \times 10^{-4}$ M). ε/dm^3 mol^{-1} cm^{-1}: 7.8 × 10^2; 7.6 x 10^2. HRMS (MALDI-TOF/TOF) *m*/z: $[M + H]^+$ calculated for $C_{21}H_{26}FeN_5O$, 416.1174; found 416,1171.

*N*9-Ferrocenoyl-N^6-(2-*iso*pentenyl)adenine (**4-N9**): resinous solid (182.1 mg, 44% isolated yield (1.0 mmol, 203.2 mg 2-*iso*pentenyladenine), mp > 200 °C. When isolated from the reaction mixture, N9 was additionally purified on a column with the eluent dichloromethane/acetone 10:0.9.

^{1}H NMR (400 MHz, $CDCl_3$, 25 °C) δ/ppm: 8,50 (s, 1H, C2-H), 8.37 (s, 1H, C8-H), 6.00 (s, broad, 1H, NH), 5.40 (t, 1H, CH=), 5.09 (pt, 2H, Cα-H), 4.70 (pt, 2H, Cβ-H), 4.28 (s, 5H, Cp-H), 4.25 (s, 2H, CH_2), 1.76 (s, braod, 6H, 2xCH_3). $^{13}C\{^1H\}$ NMR (100 MHz, $CDCl_3$, 25 °C) δ/ppm: 169.23 (FcCO), 154.91 (C6), 154.47 (C2), 148.61 (C4), 138.73 (C8), 137.01 (C=), 120.04 (C5), 119.98 (CH=), 73.51 (Cβ), 72.53 (Cα), 70.84 (Ci), 69.97 (Cp), 38.81 (CH_2), 25.69 (CH_3), 18.02 (CH_3). IR (CH_2Cl_2) ν_{max}/cm^{-1}: 3424 (w, NH), 3126 and 3048 (w C-H), 2975, 2926 (w C−H and =C−H, Fc), 1700 (s, C=O), 1615 (s, stretching of purine ring), 1500-1200 (C=C and C=N stretching). UV–vis

(CH_2Cl_2, 25 °C) λ/nm: 472; 347. ε/dm^3 mol^{-1} cm^{-1}: 8.5 × 10^2; 1.8 × 10^3. HRMS (MALDI-TOF/TOF) *m*/z: [M + H]$^+$ calculated for $C_{21}H_{26}FeN_5O$, 416.1174; found 416.1171.

*N*7-Ferrocenoyl-N^6-benzylaminopurine (**5-N7**); NMR spectra in agreement with those reported earlier[1]: ^{1}H NMR (400 MHz, $CDCl_3$) δ/ppm: 8.82 (s, 1H), 8.64 (s, 1H), 7.68 (s, 1H), 7.42 (d, 2H), 7.35 (t, 2H,), 7.28 (dd, 1H), 4.96 (pt, 2H), 4.86 (d, 2H), 4.75 (pt, 2H), 4.27 (s, 5H). ^{13}C{^{1}H} NMR (100 MHz, $CDCl_3$) δ/ppm: 171.28, 160.45, 155.50, 151.35, 144.34, 138.39, 128.71, 127.62, 127.41, 110.25, 73.84, 72.24, 71.42, 71.09, 44.94.

*N*9-Ferrocenoyl-N^6-benzylaminopurine (**5-N9**); NMR spectra in agreement with those reported earlier[1]: ^{1}H NMR (400 MHz, $CDCl_3$) δ/ppm: 8.52 (s, 1H), 8.31 (s, 1H), 7.41 (t, 2H), 7.35 (t, 2H), 7.28 (dd, C4′-H), 6.45 (s, 1H), 5.08 (pt, 2H), 4.89 (s, 2H), 4.71 (pt, 2H), 4.28 (s, 5H). ^{13}C{^{1}H} NMR (100 MHz, $CDCl_3$) δ/ppm: 169.19, 154.98, 154.47, 148.87, 138.97, 138.25, 128.72, 127.76, 127,59, 120.09, 73.54, 72.51, 71.46, 70.85, 44.63.

*N*7-Ferrocenoyl-6-di-*tert*-butoxycarbonyladenine (**6-N7**) was not isolated from the reaction mixture. ^{1}H NMR (400 MHz, DMF, 25 °C) δ/ppm: 9.57 (s, 1H, C2-H), 9.45 (s, 1H, C8-H), 5.27 (pt. 2H, Cα-H), 5.15 (pt, 2H, Cβ-H), 4.75 (s, Cp-H), 1.60 (s, 20H).

*N*9-Ferrocenoyl-N^6-di-*tert*-butoxycarbonyladenine (**6-N9**): red crystals (358.7 mg, 80% isolated yield (1.0 mmol, 336.4 mg of N^6-di-*tert*-butoxycarbonyladenine), mp > 200 °C. N9 isomer was isolated from the reaction mixture and purified by column chromatography with the eluent dichloromethane/ethyl acetate 10:0.5 – 10:1.

^1H NMR (400 MHz, $CDCl_3$, 25 °C) δ/ppm: 8.98 (s, 1H, C2-H), 8.70 (s, 1H, C8-H), 5.08 (pt. 2H, Cα-H), 4.75 (pt, 2H, Cβ-H), 4.31 (s, Cp-H), 1.49 (s, 20H). $^{13}C\{^1H\}$ NMR (100 MHz, $CDCl_3$, 25 °C) δ/ppm: 168.59 (FcCO), 153.40 (C2), 152.51 (C4), 151.19 (C6), 150.42 (C=O, Boc), 143.35(C8), 129.34 (C5), 84.17 (Cq, Boc), 73.88 (Cβ), 72.46 (Cα), 70.97 (Cp), 70.93 (Ci), 27.82 (CH_3). IR (CH_2Cl_2) ν_{max}/cm^{-1}: 2982 and 2931 (w C−H and =C−H, Fc), 1786 and 1757 (s and w C=O, Boc), 1717 (s and w C=O), 1597 (w stretching of purine ring), 1500-1200 (C=C and C=N stretching). UV–vis (CH_2Cl_2, 25 °C) λ/nm: 476; 351. $\varepsilon/dm^3\ mol^{-1}\ cm^{-1}$: 5.9×10^2; 1.1×10^3. HRMS (MALDI-TOF/TOF) *m*/z: $[M]^+$ calculated for $C_{26}H_{29}FeN_5O_5$, 547.1518; found 547.1500.

*N*7-Ferrocenoyl-purine (**purine**): red crystals (139,4 mg, 42% isolated yield (1.0 mmol, 120.2 mg of purine), mp > 200 °C. N7 isomer was isolated from the reaction mixture and purified by column chromatography with the eluent dichloromethane/acetone 7:1.

^1H NMR (400 MHz, DMSO-d_6, 25 °C) δ/ppm: 9.45 (s, 1H, C2-H), 9.43 (s, 1H, C6-H), 9.13 (s, 1H, C8-H), 5.15 (pt, 2H, Cα-H), 4.83 (pt, 2H, Cβ-H), 4.37 (t, 5H, Cp-H). $^{13}C\{1H\}$ NMR (100 MHz, DMSO-d_6, 25 °C) δ/ppm: 169.24 (FcCO), 160.93 (C4), 154.70 (C8), 148.78 (C6), 144.76 (C2), 124.45 (C5), 73.89 (Cβ), 72.11 (Cα), 71.09 (Cp), 69,97 (Ci). IR (CH_2Cl_2) ν_{max}/cm^{-1}: 3129 and 3054 (s, C−H and =C−H, Fc), 1699 (s, C=O), 1587 (s, −C=N, stretching of purine ring), 1500-1200 (C=C and C=N stretching). UV–vis (CH_2Cl_2, 25 °C) λ/nm: 472.4; 354.4. $\varepsilon/dm^3\ mol^{-1}\ cm^{-1}$: 4.5×10^2; 8.3×10^2. HRMS (MALDI-TOF/TOF) *m*/z: $[M]^+$ calculated for $C_{16}H_{12}FeN_4OH$, 332.0360; found 332.0369.

1.3. In situ ^{1}H NMR monitoring of the reaction between adenine anions and FcCOCl

The *N*9/*N*7 product ratio was determined quantitatively by integration of the respective signals in ^{1}H NMR spectra of the reaction mixture (representative spectra in Figure S1 and S2). ^{1}H NMR spectra were recorded over 30 minutes. All spectra were recorded in *N*,*N*-dimethylformamide-d_7 (DMF-d_7, > 99.5 atom % D, Sigma-Aldrich, Saint Louis, USA) or in DMF. In the latter case, the DMSO-d_6 was used as a deuterated solvent in capillary, and the DMF signal was suppressed, where available, by the presaturation options (PRESAT or WET), as included in the OpenVnmrJ, Version 2.1.A (University of Oregon).

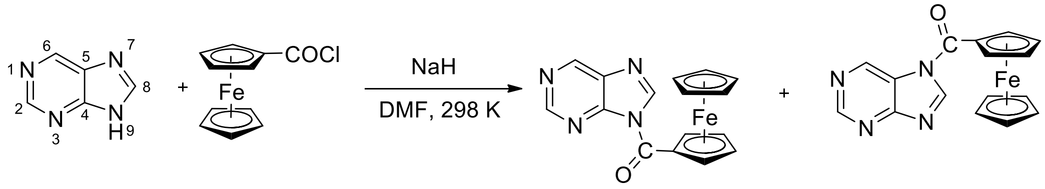

purine **purine-N9** (29%) **purine-N7** (71%)

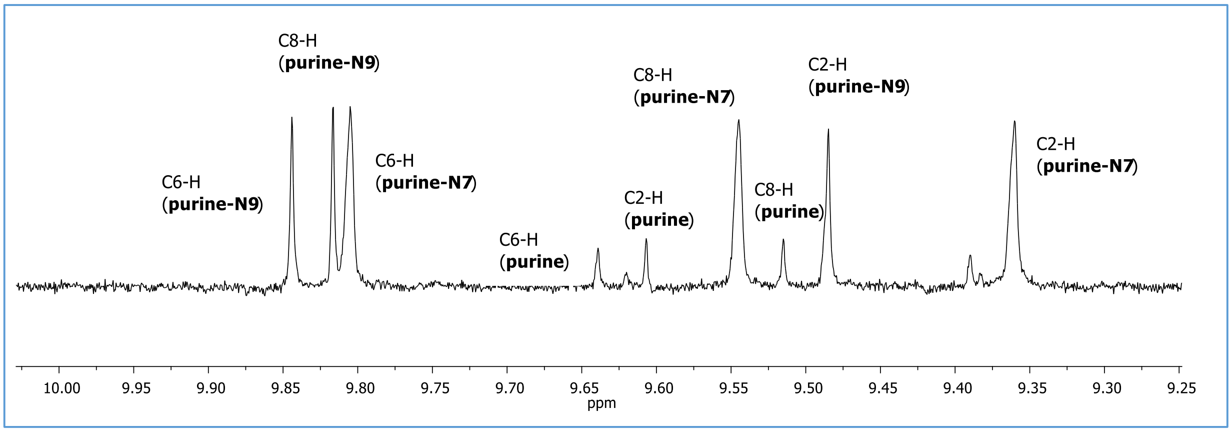

Figure S1: ^{1}H NMR spectrum (downfield region) of the reaction mixture (purine anion and FcCOCl) in DMF, taken after 20 minutes. The DMF signal was suppressed by the presaturation option (PRESAT), and DMSO-d_6 was used as a deuterated solvent in capillary.

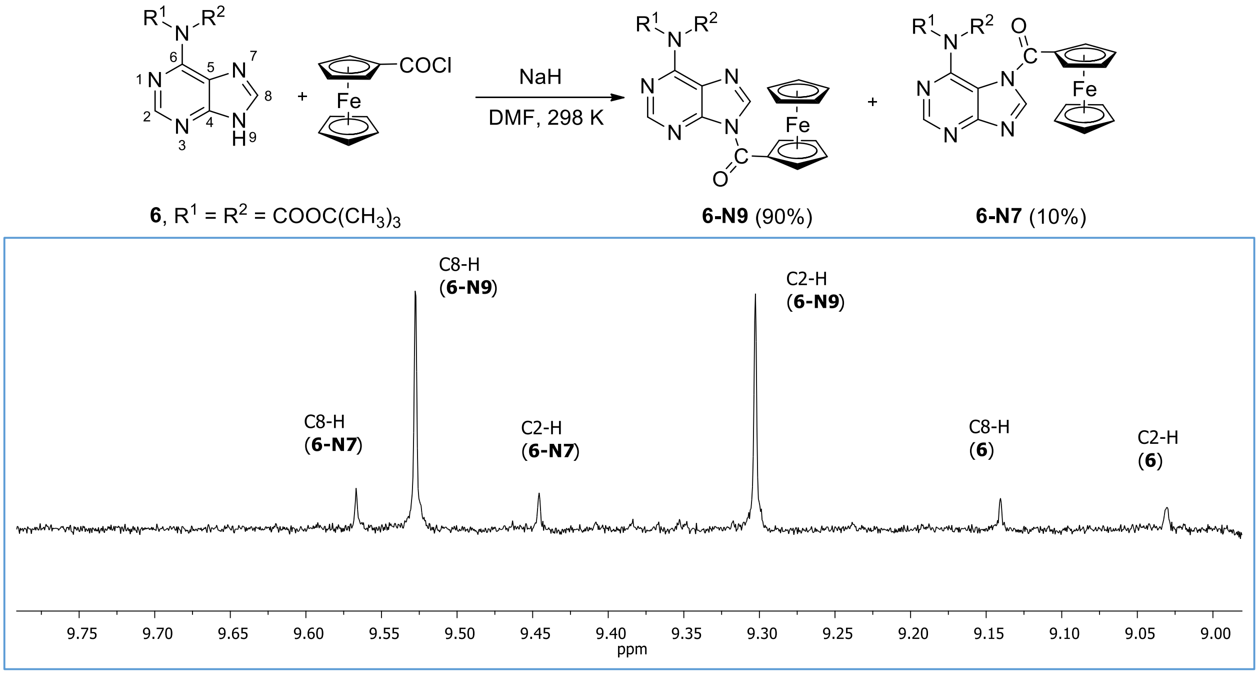

Figure S2: ^{1}H NMR spectrum (downfield region) of the reaction mixture (adenine anion **6** and FcCOCl) in DMF, taken after 30 minutes. The DMF signal was suppressed by the presaturation option (PRESAT), and DMSO-d_6 was used as a deuterated solvent in capillary.

1.4. Selected ^{1}H NMR spectra of the reaction mixture (regiospecific mode)

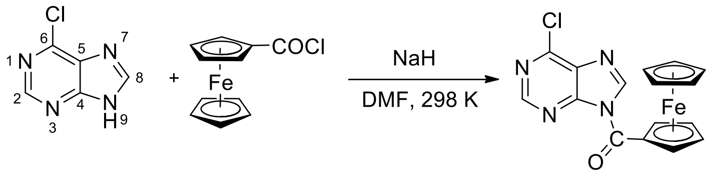

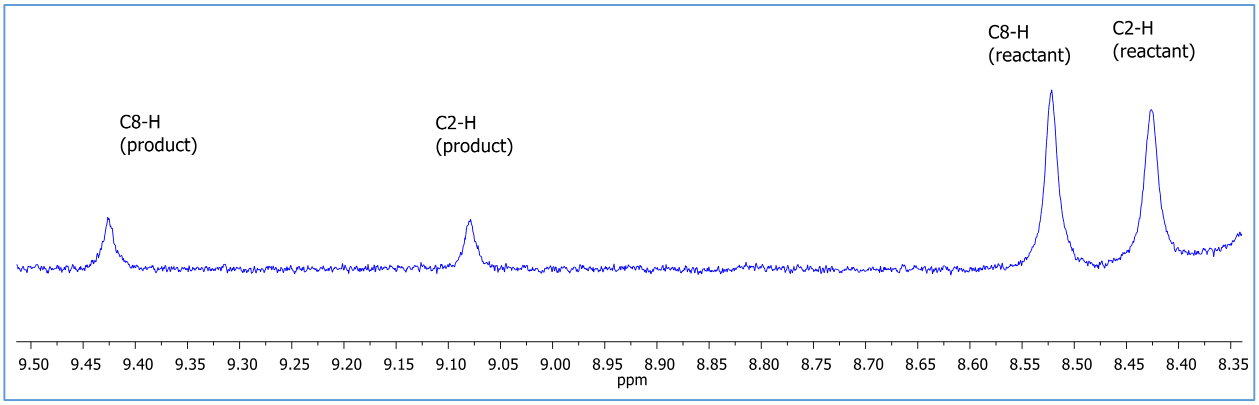

Figure S3: ^{1}H NMR spectrum (downfield region) of the reaction mixture (6-chloroadenine anion and FcCOCl) in DMF, taken after 10 minutes. The DMF signal was suppressed by the presaturation option (PRESAT), and DMSO-d_6 was used as a deuterated solvent in capillary.

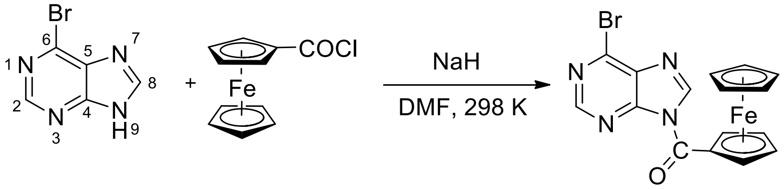

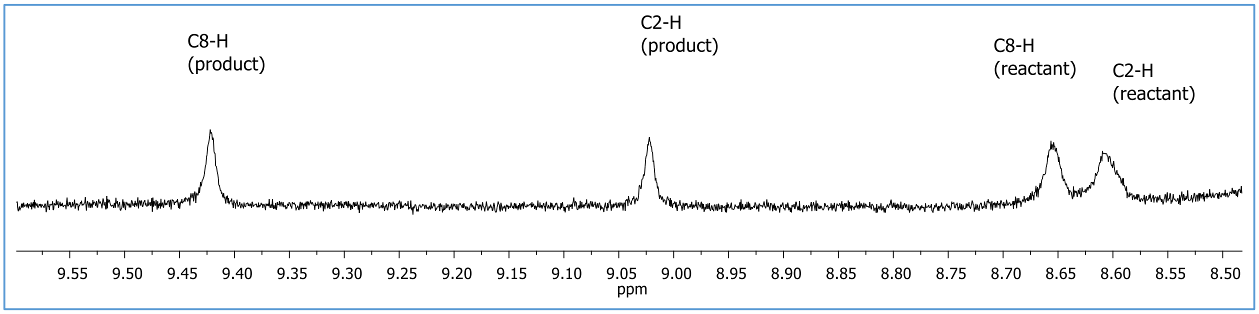

Figure S4: ^{1}H NMR spectrum (downfield region) of the reaction mixture (6-bromoadenine anion and FcCOCl) in DMF, taken after 10 minutes. The DMF signal was suppressed by the presaturation option (PRESAT), and DMSO-d_6 was used as a deuterated solvent in capillary.

2. Computational part

2.1. Computational techniques and methods

Geometries were fully optimized on the B3LYP level of theory, as implemented in Gaussian 16 software package.[2] Basis set for optimization was Pople's 6-31+G(d) on non-metal atom centers, while Stuttgart-Dresden (SDD) basis set with effective core potential (ECP) was used for Fe.[3] This DFT level has proven successful for organometallic compounds and are well suited for the description of their structures and energies.[4]

Gibbs energies of solvation were determined using the SMD model[5] at the B3LYP/6-31+G(d)//B3LYP/6-31+G(d) level. The solvent relative permittivity of $\varepsilon = 37.22$ (*N*,*N*-dimethylformamide, DMF) was used.

IRC (intrinsic reaction coordinate) calculations were performed at the corresponding level to identify the minima connected through the transition state. The initial geometries used were that of the corresponding transition structures, and the paths were followed in both directions from that point. This method verified that a given transition state connected the presumed energy minimum structures.

Nucleophilicity (Fukui index f^-) of nitrogen atom A in purine anion (of N electrons) was calculated according to the equation 1.

$$f_A^- = P_A(N) - P_A(N-1) \qquad (1)$$

where P stands for the population (or charge) of nitrogen atom A in respective purine anion. Natural population analysis and atomic charge assignments (NPA and CHelpG schemes) were calculated at the (U)B3LYP/6-31+G(d) level using the NBO Version 3.1,[6] as implemented in Gaussian 16. Conformational search was done using CREST[7,8], a part of xtb[9], using GFN2-xTB method[10].

2.2. Calculated energies and barriers

Table S1: B3LYP/6-31+G(d)/SDD calculated Gibbs free energies for reactants and corresponding transition state structures for reaction between purine anions and FcCOCl (*N*7-ferrocenoylation). Solvation energies calculated at the SMD-B3LYP/6-31+G(d)/SDD level in DMF (ε = 37.22) as a model solvent.

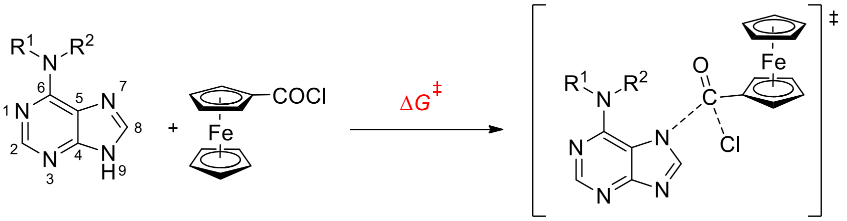

Reactant			Transition state structure			$\Delta G^{\ddagger}$ (kJ/mol)[b]
	G_{298} (Hartree)	ΔG_{solv} (kJ/mol)		G_{298} (Hartree)	ΔG_{solv} (kJ/mol)	
**purine**[a]	-411.374366	-289.9	**purine-TS$_{N7}$**	-1495.168160	-248.8	106.4
**1** $R^1 = R^2$ = H	-466.726598	-265.3	**1-TS$_{N7}$**	-1550.522334	-261.5	111.0
**2** R^1 = Me, R^2 = H	-506.008991	-260.2	**2-TS$_{N7}$**	-1589.802595	-263.1	110.0
**3** $R^1 = R^2$ = Me	-545.287581	-252.7	**3-TS$_{N7}$**	-1629.067086	-258.2	144.1
**4** R^1 = $CH_2CHC(CH_3)_2$, R^2 = H	-661.662098	-264.4	**4-TS$_{N7}$**	-1745.752028	-269.5	117.1
**5** R^1 = CH_2Ph, R^2 = H	-736.998429	-265.7	**5-TS$_{N7}$**	-1820.786674	-271.2	121.2
**6** $R^1 = R^2$ = $COOC(CH_3)_3$	-1158.168445	-249.7	**6-TS$_{N7}$**	-2241.940467	-257.8	132.3
**FcCOCl**	-1083.822441	-46.9	-	-	-	-

[a]The anion derived from the parent 9*H*-purine structure. [b] Gibbs free energy of activation calculated as $[(G_{TS}+\Delta G_{solv}) - ((G_{reactant}+\Delta G_{solv}) + (G_{FcCOCl}+\Delta G_{solv}))] - \Delta\Delta G_{solv}$, where the latter term (≈ 10 kJ/mol) is a correction term to evaluate the effect of the loss of translation degrees of freedom in solution on the Gibbs activation energy in bimolecular reaction, as reported by Ardura et al.[11]

2.3. S_N2-type and alternative reaction mechanisms for *N*7-ferocenoylation of adenines

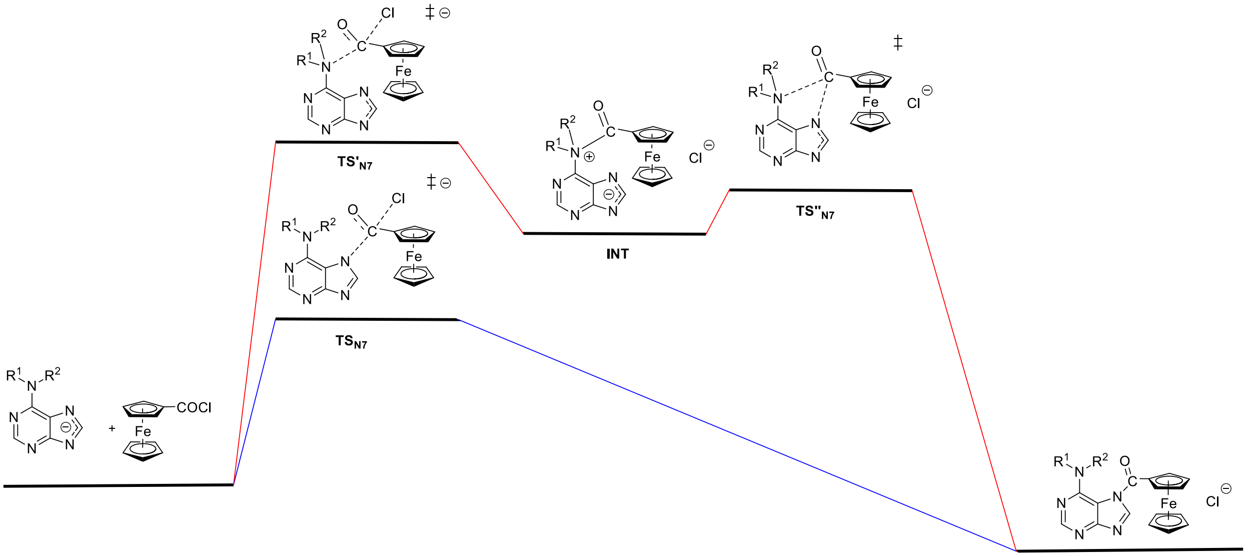

Scheme S1: One step S_N2-type *N*7-ferocenoylation of adenines via **TS**$_{\mathbf{N7}}$ (blue line) and two step reaction which includes quaternary intermediate **INT** and carbonyl group shift via **TS''**$_{\mathbf{N7}}$ (red line). The latter alternative mechanism may be applicable for **3** ($R^1 = R^2 =$ Me); see the main text.

2.4. Calculation of Sterimol and % V_{Bur} parameters

Boltzmann-weighted subparameters B_1, B_5, and L_0 are obtained as follows: first, conformational ensemble is generated, geometry optimization and energy calculation for each conformer is then performed using either semiempirical calculations and/or DFT. Sterimol parameters are computed for each conformer, while the Boltzmann-weighted parameters are obtained by summing multiplication products of conformers Sterimol parameter with weight of each conformer, which is calculated from corresponding energies.

Sterimol was used to describe substituents starting with amino moieties[12], in two different procedures. First approach was to compute Sterimol parameter for the entire substituent, while the other approach was to divide amino substituent to two sub-units, and compute Sterimol parameter for each sub-unit. Better results were obtained when the latter approach was used.

Usage of Sterimol program (wSterimol) is straightforward, but some limitations exist. Namely, only Gaussian input (.com and .gjf) and output (.out and .log) files are expected file formats. Thus, a variant, SteriXYZ was written and implemented as a web-page eliminating installation with more commonly used .xyz format as input[13].Additional feature involves automatic reading and solving of multiple conformers/optimization steps/molecular dynamic snapshots contained in the same .xyz file. This feature was implemented for automatic processing of CREST files, when conformational sampling was performed by the xtb program package using GFN2-xTB method. Useful visualization of calculated parameters are also present[14]. Example of graphical representation of Sterimol parameters can be seen in graphical abstract of this paper.

Additionally, buried volume (%VBur) is calculated using similar approach as SambVca 2.1.[15,16] For this, our own implementation was used.[13,14] For calculation of buried volume only

definition of reaction centre is needed, as compared to SambVca. Ignoring reaction center radius, target radius of 3.5 ångströms, mesh spacing value of 0.1 ångströms, and Bondi atomic radii scaled by 1.17 are used for the calculation. Hydrogen atoms are included in calculation. Boltzmann averaging was performed at 298.15 K on conformers obtained from CREST at GFN2-xTB level of theory.

Table S2: Calculated Sterimol parameters for Boltzmann-averaged purine anion conformers at CREST GFN2-xTB level of theory.

	C6-**R**			R = **R₁**-N-R²			R = R¹-N-**R²**		
Structure	L_0	B_1	B_5	L_0	B_1	B_5	L_0	B_1	B_5
**purine**	2.173	1.090	1.090	-	-	-	-	-	-
**1**	2.921	1.550	1.973	2.095	1.090	1.959	2.095	1.090	1.959
**2**	4.080	1.550	3.139	3.138	1.700	2.125	2.095	1.090	1.959
**3**	4.072	1.550	3.164	3.138	1.700	2.125	3.138	1.700	2.125
**4**	5.709	1.550	5.885	5.047	1.700	5.429	2.095	1.090	1.959
**5**	5.953	1.550	6.627	5.069	1.700	6.102	2.095	1.090	1.959
**6**	7.028	1.550	6.467	6.148	1.700	4.764	6.148	1.700	4.764
**FcCO-N**[a]				6.045	1.810	6.775			

[a] N7-ferrocenoyl purine

Table S3: Calculated % V_{Bur} (using parameters from SambVca 2.1) and minimal distance of reaction center to substituent (C6-R). Boltzmann-averaged purine anion conformers at CREST GFN2-xTB level of theory.

	**% V_{Bur}**		**Distance**	
**Structure**	**N7**	**N9**	**N7**	**N9**
**Purine**	43.27	42.38	2.926	4.399
**1**	45.96	42.36	2.769	4.628
**2**	48.68	42.37	2.433	4.501
**3**	51.03	42.39	2.112	4.300
**4**	51.67	42.49	2.395	4.396
**5**	54.21	42.51	2.315	4.429
**6**	60.62	42.94	2.608	4.291
**FcCO-N**[a]	72.71			

[a]N7-ferrocenoyl purine

2.5. Optimized coordinates of reactants and transition states

FcCOCl

Sum of electronic and thermal Free Energies= -1083.822441

23

Fe 0.861391 0.152325 -0.040055
C -1.066465 0.858208 0.130542
C -0.198534 1.575249 1.032362
C 0.790696 2.226149 0.250352
C 0.554215 1.915546 -1.126980
C -0.582908 1.069164 -1.209843
C 0.890546 -1.881370 0.399112
C 1.376678 -1.658027 -0.925026
C 2.522729 -0.809026 -0.834651
C 2.741914 -0.507642 0.544159
C 1.731844 -1.170587 1.307939
H -0.299015 1.594356 2.108854
H 1.605031 2.828220 0.632870
H 1.158285 2.244009 -1.963077
H -1.013249 0.650130 -2.108077
H 0.012198 -2.456581 0.664029
H 0.935984 -2.041498 -1.836248
H 3.104711 -0.434491 -1.667233
H 3.519047 0.134763 0.938554
H 1.607730 -1.120759 2.382087
O -2.555534 -0.107419 1.720109
C -2.179148 0.034015 0.592154
Cl -3.063060 -0.847607 -0.754818

*****

purine

Sum of electronic and thermal Free Energies= -411.374366

12

C 0.526812 0.577493 -0.000000
C 0.000000 -0.763444 -0.000000
C -2.093021 0.068372 0.000000
C -0.394849 1.618716 0.000000
H -3.170212 -0.103363 0.000000
H -0.088931 2.666508 0.000000
N -1.718191 1.367556 0.000000

N -1.325382 -1.023439 0.000000
N 1.900946 0.518536 -0.000000
N 1.036713 -1.645530 -0.000000
C 2.108029 -0.812335 -0.000000
H 3.118711 -1.215827 -0.000000

*****

1
Sum of electronic and thermal Free Energies= -466.726598

14

C -0.000000 0.554692 0.000000
C -1.035396 -0.420878 0.000000
C 0.503937 -2.058201 -0.000000
C 1.323321 0.093616 -0.000000
H 0.755224 -3.119946 -0.000000
N 1.576046 -1.227031 -0.000000
N -0.789967 -1.760926 -0.000000
N -0.547315 1.816818 0.000000
N -2.238537 0.221227 0.000000
C -1.864506 1.525107 0.000000
H -2.610741 2.316754 0.000000
N 2.391179 0.962113 -0.000000
H 2.221521 1.955468 -0.000000
H 3.330011 0.596296 -0.000000

*****

2
Sum of electronic and thermal Free Energies= -506.008991

17

N 2.643845 -0.090790 0.076950
C 2.304349 -1.403031 0.042214
H 3.069034 -2.176077 0.075241
N 0.996132 -1.727125 -0.033041
C 0.417122 -0.480439 -0.049874
C -0.918510 -0.055556 -0.102942
N -1.951083 -0.979562 -0.213170
N -1.206022 1.256054 -0.072195
C -0.156862 2.115560 -0.010498
H -0.436198 3.170085 0.006905
N 1.143236 1.852179 0.035591

C 1.425740 0.520365 0.019934
C -3.301996 -0.634248 0.185944
H -3.626788 0.255634 -0.359426
H -3.968655 -1.467678 -0.065331
H -3.397544 -0.414827 1.264149
H -1.641655 -1.918343 0.010838

*****

3
Sum of electronic and thermal Free Energies= -545.287581

20

N 2.813972 -0.398763 -0.021547
C 2.263284 -1.630845 0.072023
H 2.887974 -2.520888 0.106934
N 0.921272 -1.731013 0.120837
C 0.538537 -0.406455 0.047857
C -0.704978 0.263165 0.070643
N -1.941468 -0.377968 0.209830
N -0.719087 1.609470 0.002194
C 0.462849 2.262263 -0.069058
H 0.379860 3.349440 -0.114982
N 1.690475 1.762967 -0.096753
C 1.714856 0.405766 -0.036177
C -2.096090 -1.738837 -0.282772
H -1.169843 -2.295712 -0.134328
H -2.912124 -2.230474 0.264792
H -2.352170 -1.754824 -1.360644
C -3.144432 0.427616 0.110429
H -3.015229 1.363408 0.654943
H -3.398863 0.680699 -0.936477
H -3.979898 -0.140523 0.540159

*****

4
Sum of electronic and thermal Free Energies= -661.962098

27

C 3.301475 -2.061058 -0.082048
N 4.099913 -0.965867 -0.128711
H 3.726523 -3.061062 -0.133364
C 1.889937 -0.509267 0.057620

C 3.195321 0.050327 -0.040770
N 1.968523 -1.881222 0.028219
C 0.807596 0.377210 0.143465
C 2.313970 2.116135 0.028210
H 2.441945 3.199439 0.016747
N 1.024446 1.702088 0.119941
N 3.423368 1.392155 -0.052352
N -0.491932 -0.105301 0.283502
H -0.554650 -1.096171 0.073485
C -1.635383 0.702236 -0.118106
H -1.751732 0.735514 -1.213724
H -1.411074 1.727809 0.196141
C -2.886833 0.218936 0.563588
H -2.793419 0.162235 1.649629
C -4.058781 -0.147907 0.014990
C -4.360383 -0.154437 -1.466196
H -4.737925 -1.139366 -1.778811
H -5.150043 0.573129 -1.708250
H -3.489471 0.083480 -2.081586
C -5.212932 -0.596533 0.884200
H -4.952061 -0.577160 1.947962
H -6.095390 0.045471 0.738748
H -5.526852 -1.620139 0.629109

*****

5
Sum of electronic and thermal Free Energies= -736.998429

27

C -3.188245 0.005507 0.452884
C -3.528760 -1.910090 -0.375941
C -2.099719 -0.368850 -0.383904
H -4.021337 -2.854905 -0.594408
N -4.107167 -1.001156 0.447913
N -2.328238 -1.614685 -0.917140
N -3.230377 1.189915 1.123865
N -1.064556 1.699129 0.160095
C -2.159632 1.947088 0.923973
C -1.034024 0.533514 -0.505594
H -2.141187 2.906468 1.442577
C 1.299149 0.958422 -1.272644
H 1.676937 1.127767 -2.291858
H 1.065144 1.940101 -0.845135
C 2.397990 0.295767 -0.449408

C 3.734202 0.356659 -0.869606
C 2.105641 -0.366460 0.752808
C 4.758124 -0.218016 -0.109453
H 3.975248 0.856986 -1.806854
C 3.124743 -0.942918 1.514445
H 1.073536 -0.429627 1.085571
C 4.456006 -0.870880 1.088645
H 5.787872 -0.162680 -0.457633
H 2.877265 -1.451701 2.443607
H 5.247435 -1.324483 1.681554
N 0.045572 0.240272 -1.329593
H 0.059600 -0.720718 -1.650630

*****

6
Sum of electronic and thermal Free Energies= -1158.168445

44

C -1.625833 2.785953 -1.788129
N -1.248848 1.507455 -1.576497
H -2.182563 3.056750 -2.682354
C -0.629026 2.990050 0.059013
C -0.593412 1.615996 -0.378333
C 0.060748 0.722251 0.461621
C 0.531285 2.439639 1.904674
H 1.002085 2.734775 2.841971
N -1.298124 3.725594 -0.869798
N -0.058950 3.410283 1.206499
N 0.619077 1.126459 1.606436
N 0.193459 -0.683930 0.143082
C 1.474670 -1.260857 0.065740
O 1.732915 -2.419171 0.320535
C -0.935985 -1.497543 -0.052665
O -0.929031 -2.554391 -0.650719
O 2.359037 -0.327741 -0.336837
O -2.006104 -0.923333 0.524905
C 3.804915 -0.559306 -0.262488
C 4.216963 -0.905547 1.173993
C 4.215918 -1.645002 -1.265350
C 4.376340 0.802288 -0.672639
H 3.821471 -0.157038 1.868943
H 3.845551 -1.889036 1.470193
H 5.311952 -0.906114 1.247255
H 3.869647 -1.381821 -2.271536

H 5.310275 -1.727271 -1.289220
H 3.792902 -2.613830 -0.991766
H 5.472421 0.765162 -0.674700
H 4.032138 1.076359 -1.675657
H 4.049481 1.580998 0.023898
C -3.373483 -1.350884 0.193844
C -3.592626 -1.321951 -1.323864
C -3.641128 -2.732993 0.801909
C -4.224258 -0.276151 0.878742
H -3.226132 -0.374876 -1.734142
H -3.069467 -2.142542 -1.820253
H -4.666505 -1.412506 -1.532751
H -3.452166 -2.718593 1.882018
H -4.691588 -3.008037 0.640595
H -3.002626 -3.491461 0.343278
H -5.289596 -0.495864 0.738416
H -4.011803 -0.243865 1.953204
H -4.006844 0.709202 0.455075

*****

purine-TS-N7
Sum of electronic and thermal Free Energies= -1495.168160
Imaginary frequencies: -106.9388

35

Fe 2.467417 -0.485557 -0.192329
C 0.470073 0.054253 -0.321113
C 0.972321 -0.299765 -1.616464
C 1.418128 -1.651269 -1.561228
C 1.198163 -2.134986 -0.233195
C 0.614682 -1.084155 0.531890
C 4.011329 0.804697 -0.705956
C 3.496457 1.140107 0.583959
C 3.660943 0.003844 1.434387
C 4.277579 -1.035867 0.670123
C 4.493887 -0.540969 -0.653988
H 1.006892 0.365363 -2.468404
H 1.870557 -2.205415 -2.374517
H 1.454992 -3.119844 0.136973
H 0.346182 -1.119241 1.577776
H 3.996897 1.444523 -1.579268
H 3.007465 2.067412 0.853466
H 3.331172 -0.067765 2.462946
H 4.508742 -2.033843 1.021989

H 4.918208 -1.098107 -1.480320
C -0.065281 1.404615 -0.018826
C -2.760831 1.416515 -1.390164
N -2.145082 0.843617 -0.336186
Cl -0.208952 1.686848 1.869541
O 0.067870 2.379243 -0.710453
H -2.248472 2.169630 -1.980677
C -4.254420 0.092437 -0.692632
C -3.092987 -0.028536 0.144938
C -3.160557 -0.924451 1.206883
C -5.301437 -1.459358 0.556219
H -6.187945 -2.062870 0.750546
N -4.019293 1.022714 -1.665137
N -5.372272 -0.630897 -0.487692
N -4.274536 -1.649531 1.412809
H -2.338311 -1.069718 1.905895

*****

1-TS-N7
Sum of electronic and thermal Free Energies= -1550.522334
Imaginary frequencies: -112.1076

37

Fe 2.542614 -0.429126 0.364372
C 0.552249 0.123742 0.191514
C 0.665011 -1.305043 0.238212
C 1.275801 -1.655022 1.474996
C 1.547296 -0.448692 2.193844
C 1.101819 0.649012 1.403636
C 3.685255 -1.122570 -1.225284
C 3.542453 0.297018 -1.302562
C 4.096320 0.862694 -0.112999
C 4.582287 -0.207961 0.701248
C 4.327490 -1.435793 0.014001
H 0.326708 -1.979350 -0.536153
H 1.514857 -2.659378 1.802100
H 2.032772 -0.377414 3.159425
H 1.174287 1.697471 1.654346
H 3.328938 -1.836086 -1.957496
H 3.047183 0.844554 -2.094303
H 4.102608 1.914684 0.142340
H 5.032149 -0.109273 1.681605
H 4.550947 -2.429590 0.382198
C -0.023340 0.841709 -0.969505

C -2.489257 1.817110 0.882245
N -2.044534 0.942261 -0.045126
Cl 0.308257 2.709384 -0.872591
O -0.200569 0.378866 -2.069328
H -1.896789 2.683565 1.156836
C -4.077573 0.417291 0.813709
C -3.064319 0.012544 -0.101346
C -3.302681 -1.144358 -0.861776
C -5.334192 -1.343156 0.218615
H -6.248399 -1.928908 0.318943
N -3.691687 1.572812 1.434209
N -5.236164 -0.266883 0.989549
N -4.453751 -1.818326 -0.691450
N -2.387225 -1.661985 -1.766333
H -2.809081 -2.280273 -2.449298
H -1.714153 -0.999477 -2.142747

*****

2-TS-N7
Sum of electronic and thermal Free Energies= -1589.802596
Imaginary frequencies: -107.1879

40

Fe 2.656949 0.229544 -0.527503
C 0.692120 -0.311184 -0.145153
C 0.733403 0.940920 -0.842997
C 1.375650 0.723768 -2.094188
C 1.738774 -0.657340 -2.172988
C 1.318309 -1.296694 -0.972098
C 3.679113 1.626189 0.619625
C 3.634863 0.369006 1.297260
C 4.277794 -0.602257 0.469579
C 4.720011 0.054452 -0.721510
C 4.349609 1.432420 -0.628973
H 0.332258 1.872509 -0.468767
H 1.576599 1.479428 -2.843520
H 2.266656 -1.131641 -2.991106
H 1.456173 -2.335982 -0.711135
H 3.242442 2.551750 0.972871
H 3.145506 0.172997 2.242760
H 4.371485 -1.658024 0.689945
H 5.217493 -0.415495 -1.561057
H 4.517396 2.188724 -1.385930
C 0.108999 -0.451128 1.208667

C -2.210912 -2.291436 -0.105772
N -1.884081 -1.076458 0.387697
Cl 0.514728 -2.130474 1.981141
O -0.133891 0.454139 1.968923
H -1.557560 -3.144923 0.042906
C -3.861179 -1.115237 -0.721754
C -2.947699 -0.291063 -0.010738
C -3.298725 1.061620 0.175392
C -5.239497 0.631861 -1.003208
H -6.166224 1.051786 -1.396123
N -3.374025 -2.392851 -0.772756
N -5.032073 -0.656861 -1.236994
N -4.461398 1.512137 -0.329754
N -2.481462 1.950702 0.842088
H -1.748690 1.523472 1.399849
C -2.981515 3.236263 1.289957
H -3.335747 3.824900 0.438037
H -3.820636 3.146693 1.999085
H -2.160502 3.772954 1.777893

*****

3-TS-N7
Sum of electronic and thermal Free Energies= -1629.067086
Imaginary frequencies: -200.1902

43

Fe 3.085239 0.003104 -0.276006
C 1.053433 -0.362515 -0.446386
C 1.517802 0.399547 -1.569173
C 2.514429 -0.364911 -2.242898
C 2.674879 -1.598681 -1.537117
C 1.774341 -1.598100 -0.430618
C 3.834261 1.818446 0.395729
C 3.350254 1.046246 1.495818
C 4.086101 -0.177635 1.533132
C 5.025073 -0.164061 0.454400
C 4.869140 1.070831 -0.250231
H 1.172742 1.391329 -1.827957
H 3.074515 -0.052162 -3.115852
H 3.379085 -2.385064 -1.780010
H 1.671498 -2.367320 0.321287
H 3.450203 2.780710 0.081364
H 2.520492 1.306088 2.140366
H 3.919595 -0.991073 2.227633

H 5.710090 -0.962190 0.195307
H 5.416350 1.372353 -1.135173
C 0.013638 0.157641 0.489893
C -1.604105 -2.081534 -0.526155
N -1.603216 -0.731697 -0.382694
Cl 0.021482 -0.948880 2.142676
O -0.222473 1.324049 0.667486
H -0.677799 -2.621387 -0.680604
C -3.651511 -1.678276 -0.192483
C -2.946220 -0.439433 -0.168994
C -3.725011 0.733649 -0.029252
C -5.573478 -0.636227 0.274014
H -6.637737 -0.672693 0.508478
N -2.778906 -2.705690 -0.443744
N -4.980364 -1.797565 0.031408
N -5.040628 0.600763 0.234677
N -3.227784 2.028204 -0.141727
C -4.125640 3.129040 0.157124
H -4.686583 2.919642 1.069162
H -4.855637 3.315141 -0.651460
H -3.521065 4.033655 0.298950
C -2.287092 2.318777 -1.214243
H -1.625181 3.139559 -0.917195
H -1.673324 1.445839 -1.426580
H -2.824334 2.610487 -2.137725

*****

4-TS-N7
Sum of electronic and thermal Free Energies= -1745.752028
Imaginary frequencies: -111.7388

50

Fe 2.836459 0.151354 -0.557403
C 1.035433 -0.772383 -0.108353
C 0.806551 0.401574 -0.900362
C 1.484908 0.232568 -2.140261
C 2.139237 -1.038883 -2.118077
C 1.864325 -1.658889 -0.866068
C 3.522626 1.814857 0.478672
C 3.761128 0.628231 1.238065
C 4.603644 -0.229077 0.465569
C 4.886241 0.427294 -0.773317
C 4.217530 1.691163 -0.765410
H 0.221534 1.256702 -0.590559

H 1.520799 0.953821 -2.947359
H 2.759615 -1.449908 -2.904884
H 2.224006 -2.620134 -0.528668
H 2.890464 2.641435 0.777361
H 3.328965 0.392027 2.202030
H 4.931345 -1.219946 0.753289
H 5.474993 0.024172 -1.588192
H 4.210677 2.412651 -1.573197
C 0.489605 -0.928984 1.259867
C -1.232388 -3.359381 0.065539
N -1.271274 -2.096283 0.545759
Cl 1.293643 -2.374738 2.179246
O 0.032774 -0.038110 1.935606
H -0.388774 -4.007839 0.278070
C -3.082805 -2.663169 -0.695091
C -2.471762 -1.622573 0.053101
C -3.177789 -0.408433 0.181602
C -4.831656 -1.333805 -1.143967
H -5.794369 -1.167574 -1.628882
N -2.277455 -3.769574 -0.673620
N -4.287117 -2.530845 -1.311905
N -4.362017 -0.273279 -0.445501
N -2.708142 0.632214 0.946410
C -3.341058 1.939835 1.000604
H -4.417843 1.782677 0.873722
H -3.179458 2.354967 2.000996
C -2.846560 2.875882 -0.079244
H -2.979566 2.477611 -1.086011
C -2.273566 4.085565 0.052832
C -1.836159 4.873084 -1.163245
C -2.008265 4.782703 1.368100
H -2.047629 4.334229 -2.093134
H -2.345553 5.847881 -1.211665
H -0.756799 5.087200 -1.130823
H -0.944894 5.050915 1.455480
H -2.573598 5.724926 1.432481
H -2.270782 4.176754 2.238796
H -1.778470 0.528959 1.340264

*****

5-TS-N7
Sum of electronic and thermal Free Energies= -1820.786674
Imaginary frequencies: -115.6184

50

Fe 3.493144 0.604516 -0.472537
C 1.777713 -0.433141 0.053031
C 1.688098 0.015903 -1.306120
C 2.764149 -0.567279 -2.032838
C 3.524826 -1.372263 -1.128022
C 2.918626 -1.290087 0.157958
C 3.582614 2.678449 -0.529830
C 3.666370 2.218125 0.820193
C 4.815929 1.376662 0.929675
C 5.443734 1.315684 -0.353869
C 4.680894 2.120358 -1.256693
H 0.928328 0.682469 -1.690014
H 2.982754 -0.406854 -3.081346
H 4.422950 -1.926986 -1.369952
H 3.259373 -1.769504 1.064224
H 2.799841 3.305397 -0.937702
H 2.950313 2.419539 1.606615
H 5.127185 0.844650 1.819609
H 6.322682 0.735254 -0.606307
H 4.881051 2.256396 -2.312426
C 0.821612 -0.001883 1.099832
C -0.121793 -3.057551 1.281639
N -0.437956 -1.810697 0.867478
Cl 1.438803 -0.439809 2.837863
O 0.062073 0.932956 1.011199
H 0.687444 -3.219444 1.985830
C -1.735986 -3.435044 -0.036730
C -1.488264 -2.035941 -0.001159
C -2.331480 -1.205117 -0.766142
C -3.424755 -3.115677 -1.476246
H -4.224308 -3.514321 -2.101355
N -0.853785 -4.072742 0.791419
N -2.717918 -4.000223 -0.787017
N -3.301915 -1.767962 -1.508360
N -2.187375 0.166256 -0.785196
C -3.113760 1.055797 -1.448359
H -3.628741 0.461098 -2.210758
H -2.549854 1.842767 -1.969895
H -1.532329 0.561493 -0.116611
C -4.151721 1.718491 -0.547837
C -4.649604 2.989421 -0.869811
H -4.259192 3.509350 -1.744011
C -5.631340 3.600706 -0.083853
H -5.999306 4.589988 -0.349104
C -6.128889 2.945825 1.046965

H -6.888040 3.419190 1.665901
C -5.634020 1.680350 1.380166
H -6.009645 1.163467 2.260533
C -4.654069 1.072616 0.590414
H -4.269156 0.092895 0.857032

*****

6-TS-N7
Sum of electronic and thermal Free Energies= -2241.940467
Imaginary frequencies: -162.5017

67

Fe -3.815608 0.130824 -0.065703
C -1.925805 -0.143694 0.727283
C -2.024558 1.152581 0.121792
C -3.057930 1.871200 0.788835
C -3.602690 1.022806 1.803772
C -2.905196 -0.219720 1.767273
C -4.204553 -0.099630 -2.092216
C -4.110760 -1.376695 -1.459053
C -5.115281 -1.442596 -0.445397
C -5.831314 -0.204448 -0.450152
C -5.267533 0.626520 -1.468530
H -1.417050 1.494517 -0.704212
H -3.394197 2.872247 0.547622
H -4.424451 1.267852 2.465427
H -3.095462 -1.086291 2.383753
H -3.552502 0.267151 -2.874770
H -3.364973 -2.134308 -1.662085
H -5.269816 -2.269086 0.236442
H -6.636529 0.068994 0.220834
H -5.570545 1.639551 -1.703521
C -0.948544 -1.158137 0.255102
C 0.352886 -1.058649 3.014629
N 0.504548 -0.788842 1.698217
Cl -1.460894 -2.944313 0.967220
O -0.407110 -1.169371 -0.813514
H -0.531769 -1.572660 3.370956
C 2.233608 -0.100793 2.989990
C 1.727564 -0.159202 1.639959
C 2.552792 0.385311 0.657574
C 4.088991 0.941250 2.276588
H 5.052465 1.400723 2.492577
N 1.336906 -0.679754 3.840329

N 3.414350 0.452450 3.315530
N 3.728431 0.932406 0.979980
N 2.232849 0.420400 -0.756108
C 1.988485 1.670751 -1.350054
O 2.152059 1.952991 -2.517356
C 2.438751 -0.722781 -1.560492
O 2.237312 -0.781228 -2.752490
O 1.523882 2.515089 -0.399539
O 2.898399 -1.718049 -0.780278
C 1.451758 3.961731 -0.633754
C 2.831891 4.506174 -1.025088
C 0.384146 4.279815 -1.689052
C 1.031608 4.496483 0.739923
H 3.584102 4.186412 -0.295738
H 3.133373 4.157378 -2.015335
H 2.800662 5.603050 -1.033544
H -0.583262 3.858827 -1.391987
H 0.268651 5.367912 -1.775829
H 0.660286 3.873422 -2.664129
H 0.920472 5.586831 0.700495
H 0.077216 4.056933 1.048573
H 1.782237 4.245908 1.496619
C 3.115000 -3.073111 -1.310461
C 1.815253 -3.645585 -1.887472
C 4.251525 -3.047103 -2.340749
C 3.534807 -3.848607 -0.057032
H 1.003778 -3.565798 -1.158231
H 1.515053 -3.118585 -2.795219
H 1.969299 -4.705983 -2.126714
H 5.151305 -2.596157 -1.905581
H 4.494243 -4.074094 -2.642431
H 3.966034 -2.478385 -3.228596
H 3.748430 -4.892121 -0.317926
H 4.433392 -3.407451 0.388355
H 2.735218 -3.830203 0.690223

*****

3. References

1. Toma, M.; Božičević, L.; Lapić, J.; Djaković, S.; Šakić, D.; Tandarić, T.; Vianello, R.; Vrček., V. *J. Org. Chem*. **2019**, *84*, 12471−80.

2. Frisch, M.; Trucks, G.; Schlege, H.; Scuseria, G.; Robb, M.; Cheeseman, J.; Scalmani, G.; Barone, V.; Mennucci, B. Gaussian 16. Rev. B.01, Wallingford, CT, 2016.

3. Dolg, M.; Wedig, U.; Stoll, H.; Preuss, H. *J. Chem. Phys*. **1987**, *86*, 866-872.

4. Lapić, J.; Havaić, V.; Šakić, D.; Sanković, K.; Djaković, S.; Vrček, V. *Eur. J. Org. Chem*. **2015**, *24*, 5424-5431.

5. Marenich, A. V.; Cramer C. J.; Truhlar, D. G. *J. Phys. Chem. B* **2009**, *113*, 6378-96.

6. Reed, A. E.; Curtiss, L. A.; Weinhold, F. *Chem. Rev*. **1988**, *88*, 899-926.

7. Pracht, P.; Bohle, F.; Grimme, S. *Phys. Chem. Chem. Phys*. **2020**, *22*, 7169-7192.

8. Grimme S. *J. Chem. Theory Comput*. **2019**, *15*, 2847-2862.

9. Bannwarth, C.; Caldeweyher, E.; Ehlert, S.; Hansen, A.; Pracht, P.; Seibert, J.; Spicher, S.; Grimme, S. *WIREs Comput. Mol. Sci*. **2020**, *11*, e01493.

10. Bannwarth, C.; Ehlert, S.; Grimme, S. *J. Chem. Theory Comput*. **2019**, *15*, 1652-167.

11. Ardura, D.; López, R.; Sordo, T. L. *J. Phys. Chem. B* **2005**, *109*, 23618−23623.

12. Brethome, A. V.; Fletcher, S. P.; Paton, R. S. *ACS Catal*. **2019**, *9*, 2313−2323.

13. Github: https://github.com/DSakicLab/SteriXYZ

14. Web-page: http://sw.pharma.hr/SteriXYZ/

15. Falivene, L.; Cao, Z.; Petta, A.; Serra, L.; Poater, A.; Oliva, R.; Scarano, V.; Cavallo L. *Nat. Chem*. **2019**, *11*, 872–879.

16. SambVca 2.1 A web application to characterize catalytic pockets. Available at https://www.molnac.unisa.it/OMtools/sambvca2.1/index.html (Accessed on August 15, 2022)
